# Stage-specific gene expression during urediniospore germination in *Puccinia striiformis *f. sp *tritici*

**DOI:** 10.1186/1471-2164-9-203

**Published:** 2008-05-01

**Authors:** Yonghong Zhang, Zhipeng Qu, Wenming Zheng, Bo Liu, Xiaojie Wang, Xiaodan Xue, Liangsheng Xu, Lili Huang, Qingmei Han, Jie Zhao, Zhensheng Kang

**Affiliations:** 1 College of Plant Protection and Shaanxi Key Laboratory of Molecular Biology for Agriculture, Northwest A&F University, Yangling, Shaanxi, 712100, P.R. China; 2 College of Life Sciences, Henan Agriculture University, Zhengzhou, Henan, 450002, P.R. China; 3 College of Life Sciences, Northwest A&F University, Yangling, Shaanxi, 712100, P.R. China

## Abstract

**Background:**

*Puccinia striiformis *f. sp. *tritici *is an obligate biotrophic pathogen that causes leaf stripe rust on wheat. Although it is critical to understand molecular mechanisms of pathogenesis in the wheat stripe rust fungus for developing novel disease management strategies, little is known about its genome and gene functions due to difficulties in molecular studies with this important pathogen. To identify genes expressed during early infection stages, in this study we constructed a cDNA library with RNA isolated from urediniospores of *P. striiformis *f. sp. *tritici *germinated for 10 h.

**Results:**

A total of 4798 ESTs were sequenced from the germinated urediniospore library and assembled into 315 contigs and 803 singletons. About 23.9% and 13.3% of the resulting 1118 unisequences were homologous to functionally characterized proteins and hypothetical proteins, respectively. The rest 62.8% unisequences had no significant homologs in GenBank. Several of these ESTs shared significant homology with known fungal pathogenicity or virulence factors, such as HESP767 of the flax rust and *PMK1*, *GAS1*, and *GAS2 *of the rice blast fungus. We selected six ESTs (Ps28, Ps85, Ps87, Ps259, Ps261, and Ps159) for assaying their expression patterns during urediniospore germination and wheat infection by quantitative real-time PCR. All of them had the highest transcript level in germinated urediniospores and a much less transcript level in un-germinated urediniospores and infected wheat tissues (1–7 dpi). The transcript level of Ps159 increased at later infection stages (6–7 dpi). Our data indicated that these genes were highly expressed in germinated urediniospores and may play important roles in fungal-plant interactions during early infection stages in the wheat stripe rust fungus.

**Conclusion:**

Genes expressed in germinated urediniospores of *P. striiformis *f. sp. *tritici *were identified by EST analysis. Six of them were confirmed by quantitative real-time PCR assays to be highly expressed in germinated urediniospores.

## Background

Wheat stripe rust is one of the most important diseases of wheat throughout the world. It is a major constraint in wheat production and is a serious threat to food security worldwide [1,2]. *Puccinia striiformis *Westend f. sp. *tritici *Eriks is the causal agent of wheat stripe rust. It is an obligate biotrophic basidiomycete with an incomplete life cycle. Because of the high variability in the pathogen population, race-specific resistance in many newly developed cultivars often fails within a few years of cultivation and results in severe yield losses. In the past century, most of the *P. striiformis *f. sp. *tritici *studies had been focused on the identification of physiological races, virulence variation, and ultrastructural and histological examinations [1,3,4].

Unlike the wheat stem rust fungus, the sexual stage of *P. striiformis *f. sp. *tritici *has not been identified. Urediniospore is the most common spore form that has been observed in the wheat stripe rust fungus, which is strictly dependent on living host cells for growth and reproduction. To date, no stable transformation system has been established for *P. striiformis *f. sp. *tritici *and other *Puccinia *species. These biological characteristics make molecular and genetic studies of this important fungus relatively more difficult. Although much progresses have been achieved in researches on its genetic diversity, population structure, and evolution [5-8], there is only very limited knowledge about genes involved in the initial infection and biotrophic growth stages of the wheat stripe rust fungus. Such knowledge is critical for understanding infection mechanisms of this important pathogen and developing better disease management strategies.

Various genomic approaches, such as expressed sequence tag (EST) [9], serial analysis of gene expression (SAGE) [10], and massive parallel signature sequencing (MPSS) [11], have been widely used in genome-wide gene expression studies in various organisms. EST analysis was the first method used for rapid identification of expressed genes [9]. It has been employed to identify genes that are expressed in various tissues, cell types, or developmental stages in different organisms [12-14]. The availability of EST sequences has accelerated molecular characterization of genes of interest and provided sequence information for microarray design.

EST analyses have been conducted in a few rust fungi. To examine gene expression during infection of broad bean by *Uromyces fabae*, Hahn and his colleagues sequenced ESTs from purified haustoria. A major shift in gene expression was observed between urediniospore germination and the biotrophic growth stage [15,16]. A total of 25,558 ESTs have been generated from 13 cDNA libraries representing various stages of *Puccinia triticina*, including resting and germinated urediniospores, appressorium and haustorium formation stages during infection of a susceptible wheat cultivar, and infected leaves of a resistant wheat cultivar. While 38% unigenes matched sequences in various databases and collections, the annotation rates were low for ESTs from germinated urediniospores (4%) and appressoria (2%). Gene sets obtained from these different libraries appeared to be remarkably different, suggesting drastic reprogramming of the transcriptome during these major differentiation processes [17]. In this study, we generated a cDNA library from germinated urediniospores of *P. striiformis *f. sp. *tritici*. A total of 4798 ESTs were sequenced to generate 1118 unisequences (uniseqs). The majority of these ESTs (over 60%) had no significant homologs in GenBank, indicating that many of them may represent genes unique to *P. striiformis *f. sp. *tritici*. Several of these ESTs share significant homology with known fungal pathogenicity or virulence factors, such as HESP767 of the flax rust and *PMK1 *of the rice blast fungus. The high transcript level of six selected genes in germinated urediniospores was confirmed by quantitative real-time PCR (qRT-PCR) assays. Some of these genes highly expressed in germinated urediniospores may be important for early infection processes in *P. striiformis *f. sp. *tritici*.

## Results

### Generating ESTs of germinated urediniospores

**Figure 1:**
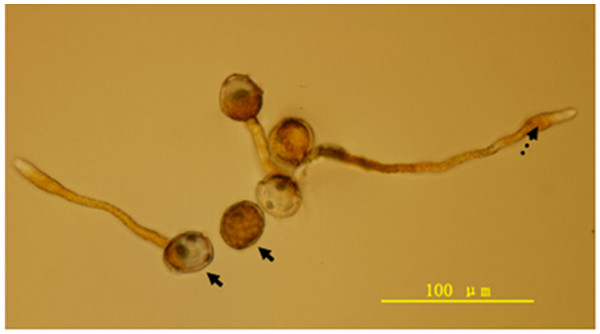
**Urediniospores and germ tubes of *P. striiformis *f. sp *tritici *(race CY32) after incubating for 10 h on plastic surface**. Solid arrowheads marked germinated and un-germinated urediniospores. Dashed arrowhead indicated the swelling of germ tubes at the tip. Bar = 100 μm.

To identify genes related to early events of urediniospore germination and differentiation, we incubated freshly harvested urediniospores of *P. striiformis *f. sp *tritici *(race CY32) in sterile distilled water in plastic plates. After incubating for 10 h at 9°C, about 60% of urediniospores attached to the plastic surface and produced long, un-branched germ tubes (Fig. 1). Some of these germ tubes displayed various degree of swelling at the tip (Fig. 1). Germinated and un-germinated urediniospores were collected at 10 h and used for RNA isolation. A directional cDNA library consisting of 6.05 × 10^5 ^primary clones was constructed with the λTripEx2 vector (Clontech). On average, about 95% of the cDNA clones had inserts longer than 0.2 kb. The insert size varied from 200–3500 bp, with an average of 750 bp. A total of 5500 random cDNA clones were sequenced from the 5'-end to obtain 4810 quality reads or ESTs.

### Data analysis and functional classification

Before clustering analysis, ESTs containing no insert sequence or insert shorter than 100 bp were removed. The remaining 4798 ESTs were aligned and assembled into 315 contigs (containing two or more ESTs) and 803 singletons. A total of 1118 unisequences (uniseqs) were submitted to dbEST at NCBI [18] under GenBank accession numbers ES321929 – ES323046. Most of the uniseqs had the insert size of 200–900 bp. Only 36 uniseqs were longer than 1000 bp. The G+C content of these ESTs ranged from 40.74% to 53.73%, with an average of 45.08%, which was similar to the G+C content of ESTs in *P. graminis *[12].

The number of individual ESTs belonging to each contig ranged from 2 to 608 [see Additional file 1]. Nine most abundant contigs contained more than 100 ESTs each, suggesting a high transcript level of the corresponding genes. Contig Ps303 was represented by 608 clones, more than any other contigs [see Additional file 2]. It is homologous to a *Saccharomyces cerevisiae *gene encoding a hypothetical protein. Five of these most abundant contigs [see Additional file 2] had no homologs in EST or protein database bases. Three other abundant contigs displayed limited homology with entries in GenBank. Contig Ps253 had weak homology with a putative secreted protein in *Ixodes scapularis*. Contig Ps28 consisting of 128 ESTs was homologous to a differentiation-related protein Inf24 from *Uromyces appendiculatus*. Inf24 was identified as infection structure protein that was highly expressed during urediniospore germination [19]. For contig Ps314, it consisted of 154 ESTs and was homologous to a predicted protein of *Kluyveromyces lactis*.

All uniseqs were subjected to similarity searches against sequences in the non-redundant protein (nr) database at GenBank using the BLASTX algorithm. InterProScan was used to analyze uniseqs with no BLASTX matches for known protein domains. The vast majority (703) of the uniseqs had no significant homolog in GenBank by BLASTX search. Among 415 uniseqs displayed similarity (E-value < 10e-5) to entries in the nr database, about 56% of them were similar to genes coding for proteins with unknown functions (not classified in Gene Ontology). The frequency of orphan sequences in *P. striiformis *f. sp. *tritici *ESTs is similar to that has been observed in other fungal EST projects [20].

Among the 415 uniseqs that exhibited similarity to entries in the non-redundant protein database at GenBank, about 40.72% shared homology to proteins from filamentous fungi while the rest were homologous to proteins from a wide variety of organisms, including yeast, bacteria, nematodes, plants, insects, and animals [see Additional file 3]. The uniseqs with significant similarity to hypothetical proteins were placed in the unclassified protein category. Based on the results from BlastX and InterProScan searches, ESTs with significant matches were categorized according to their putative functions (Table 1, for detail [see Additional file 4]).

**Table 1:** Major functional categories of *P. striiformis *ESTs from the germinated urediniospore library.

Functional category	No. of EST^a^
Cell division and chromosome structure	46
Cell signal and communication	75
Cell structure and growth	
Cytoskeletal	13
growth sporulation	409
Metabolism	
Amino Acid	49
TCA cycle/Oxidative Phosphorylation/Electron Transport	70
Lipid/Fatty Acid	16
Carbon Metabolism	70
Plant Cell Degradation	79
Transport	46
Other metabolism	15
Cell/Organism defense	
Detoxification	72
DNA repair	2
Stress response	33
Protein biosynthesis	
ribosomal protein	861
translation factors	5
tRNA synthesis	3
protein turnover	13
post-translation modification/trafficking	24
RNA synthesis	
RNA polymerases	3
transcription regulation	13
RNA processing	42
Transposon	2
Unclassified	2837

^a ^The original EST number and best hit were listed in Additional file 4 [see Additional file 4].

**Figure 2:**
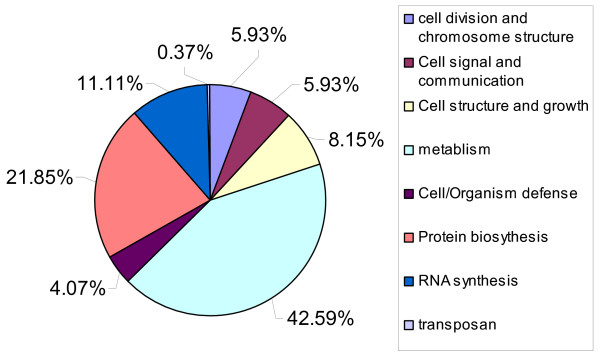
**Classification of the *P. striiformis *f. sp. *tritici *uniseqs homologous to proteins of known functions**. A total of 267 uniseqs (23.9% of 1118 uniseqs) with significant homologs in GenBank were categorized according to the functions of their homologs. Over 60% of them were involved in primary metabolism (42.59%) and protein synthesis (21.58%).

A total of 267 uniseqs were identified to be homologous to proteins with known cellular functions. Figure 2 listed the putative functions of these uniseqs and their occurrence. Over 70% of them were functionally related to primary metabolism (42.59%) and protein (21.58%) or (11.11%) RNA synthesis. About 10% were involved in cellular signaling and defense responses. The ESTs with no hits in GenBank were searched against the genome sequence of the wheat stem rust *P. graminis *[21]. Many of them had homologous sequences in the genome of the stem rust fungus (data not shown). A total of 195 uniseqs had no homologous sequences in the stem rust fungus genome. These ESTs may represent genes unique to *P. striiformis *f. sp. *tritici*.

### Putative pathogenicity or virulence factors identified in the germinated urediniospore ESTs

To date, many genes important for fungal pathogenesis have been identified in plant and human pathogens [22-24]. Homology searches revealed that several ESTs were homologous to known fungal pathogenicity or virulence factors (E-value < 1e-5), including key components of signal transduction pathways, transporters, and genes involved in infection-related morphogenesis (Table 2). For three genes related to signal transduction, Ps1728 encodes a putative adenylate cyclase that has been shown to be essential for pathogenicity in *Magnaporthe grisea*, *Ustilago maydis*, and several other fungi [25-28]. Ps259 encodes a putative Gα subunit that is similar to MagB of *M. grisea *and Gpa1 of *Cryptococcus neoformans *[29,30]. The MAP kinase encoded by Ps261 is homologous to Pmk1 of *M. grisea*, which is important for regulating plant infection processes in a number of phytopathogenic fungi [31-33].

Ps8 and Ps28 may be related to the infection structure differentiation (Table 2). Both of them were homologous to Inf24 of *U. appendiculatus *and appeared to be members of a multigene family. Inf24 is highly expressed during urediniospore germination. Microinjection of antisense oligonucleotides of Inf24 inhibits appressorium development in *U. appendiculatus *[19]. Both Ps28 and Ps228 were contigs represented by over 100 ESTs, indicating that these two genes are highly expressed in germinated urediniospores, and may have similar functions during early stages of plant infection in *P. striiformis *f. sp. *tritici*.

Contigs Ps6 and Ps159 encoded proteins homologous to gEgh16 of *Blumeria graminis *[22], which is highly expressed at early infection stages (16 h). In *M. grisea*, its homologs, *GAS1 *and *GAS2*, are important virulence factors that are involved in appressorial penetration [34]. Both *GAS1 *and *GAS2 *are specifically expressed during appressorium formation. We also identified two contigs, Ps300 and Ps5709, that were putative ATP-binding cassette (ABC) transporter genes. In *M. grise*a and other plant pathogens, several ABC transporter genes have been implicated in fungal-plant interactions, possibly by controlling the efflux of phytotoxic fungal metabolites or plant defensive compounds [35].

**Table 2:** Uniseqs with significant homology to known fungal pathogenicity factors

Uniseqs	No. of clones	Putative function	Homolog in GenBank (accession number)	E-value
Ps259	2	G protein alpha subunit	*Magnaporthe grisea MAGB *(AAB65426)	9E-47
Ps261	5	MAP kinase	M. grisea PMK1 (AAC49521)	2E-25
Ps8	14	differentiation-related protein Inf24	*Uromyces appendiculatus *Inf24 (AF155928)	8E-10
Ps28	128	differentiation-related protein Inf24	*U. appendiculatus *Inf24 (AF155928)	3E-11
Ps85	63	mycelial surface antigen precursor	*Candida albicans CSA1 *(AF080221)	1E-05
Ps238	9	secreted Cu/Zn superoxide dismutase	*C. albicans SOD *(EAL00626)	1E-13
Ps300	3	putative ABC transporter	*C. albicans RLI1 *(EAL02734)	3E-92
Ps5709	1	putative ABC transporter	*M. grisea ABC1 *(CH476837)	3E-08
Ps101	2	amino acid transporter	*Uromyces fabae AAT1 *(AJ308252)	3E-33
Ps6	4	homolog of Gas1	*M. grisea GAS1 *(AF363065)	2E-06
Ps159	19	homolog of Gas2	*M. grisea GAS2 *(AF264035)	1E-08
Ps1728	1	adenylate cyclase	*M. grisea MAC1 *(AF006827)	3E-08
Ps87	2	effector protein	*Melampsora lini *HESP-767 (DQ279883)	9E-30

We also identified an EST, Ps238 (Table 2), that encodes a putative copper- and zinc-superoxide dismutase. In some fungal pathogens, such as *Candida albicans*, superoxide dismutase is required for tolerance to oxidative stress and full virulence [36]. Contig Ps85 encoded a protein consisting of 169 amino acid residues. It is homologous a putative cell surface antigen gene in *C. albicans *[37] and contains a CFEM (Common in several Fungal Extracellular Membrane proteins) domain that is unique to filamentous ascomycetes [38]. In *M. grisea*, the CFEM domain-containing protein Pth11 is known to play an important role in pathogenesis as a receptor [39]. Pth11 is not required for appressorium morphogenesis *in vitro *but is involved in host surface recognition. The protein encoded by contig Ps85 may play a similar role in *P. striiformis *f. sp. *tritici*, and is involved in appressorium differentiation.

### ESTs homologous to known rust effectors or avirulence genes

The first Avr protein identified in rust fungi was AvrL567 of the flax rust fungus *Melampsora lini *[40]. Recently, a number of additional avirulence elicitors have been identified in the flax rust fungus [41]. In the ESTs of the wheat stripe rust fungus, we identified a candidate avirulence elicitor encoded by contig Ps87, which had significant homology with HESP767. In the flax rust fungus, HESP767 is expressed in haustoria and may function as an effector in interactions with the host plant [41]. Ps87 contained the full length open reading frame (ORF) that was predicted to encode a protein of 85 amino acid residues. It had a typical signal peptide at the N-terminus (data not shown) as predicted by SignalP 3.0 [42]. Similar to HESP767, Ps87 may function as an effector or avirulence factor during wheat infection in *P. striiformis *f. sp. *tritici*.

### Assaying the transcript level of six selected ESTs

**Figure 3:**
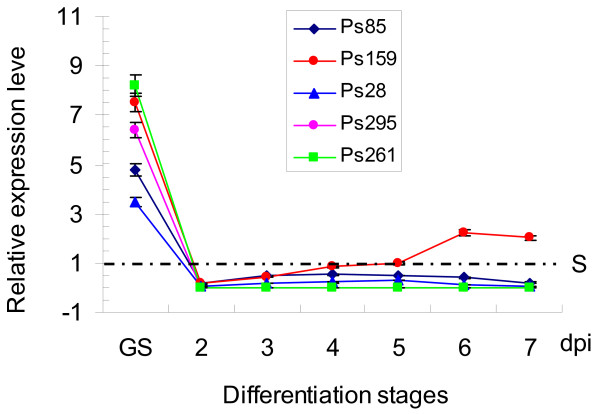
**Assays for the transcript levels of ESTs Ps28, Ps85, Ps159, Ps259, and Ps261 in *P. striiformis *f. sp. *tritici***. RNA samples were isolated from urediniospores, germinated urediniospores, or leaves of wheat cultivar Huixianhong inoculated with CY32 and collected at the indicated time points. The expression level of these genes was calculated by the comparative Ct method with the actin gene of *P. striiformis *f. sp. *tritici *as the endogenous reference for normalization. Relative quantification was computed with their expression levels in different stages in comparison to that in urediniospores. The average and standard error were calculated from three biological replicates. S, urediniospores; GS, germinated urediniospores; dpi, days post inoculation.

**Figure 4:**
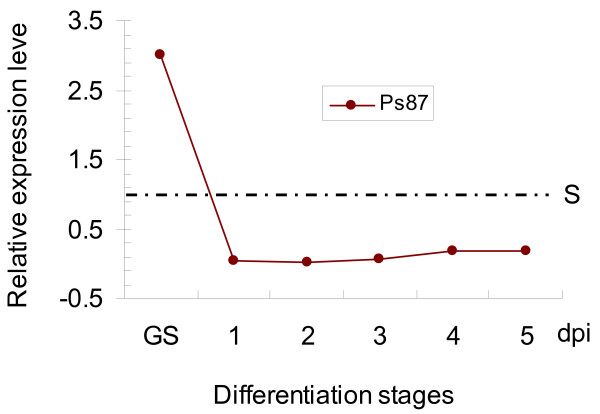
**Assays for the transcript level of Ps87 during incompatible interactions**. RNA samples were isolated from urediniospores, germinated urediniospores, or leaves of wheat cultivar Shuiyuan 11 inoculated with CY23 and collected at the indicated time points. The expression level of Ps87 was calculated by the comparative Ct method with the actin gene of *P. striiformis *f. sp. *tritici *as the endogenous reference for normalization. Relative quantification was computed with its expression levels in different stages in comparison to that in urediniospores. The average and standard error were calculated from three biological replicates. S, urediniospores; GS, germinated urediniospores; dpi, days post inoculation.

Six uniseqs, Ps28 (a differentiation-related protein), Ps85 (a CFEM domain-containing protein), Ps159 (gEgh16 homolog), Ps259 (G-alpha subunit), Ps261 (Pmk1 homolog), and Ps87 (HESP767 homolog), were selected for assaying their transcript levels during different developmental and infection stages by quantitative real-time PCR (qRT-PCR). All these six genes were expressed in urediniospores, germinated urediniospores, and infected wheat tissues harvested at 1 through 7 days post inoculation (dpi). However, their transcription levels were much higher in germinated urediniospores than in un-germinated urediniospores or infected wheat tissues (Fig. 3; Fig. 4).

Although the transcript level of these genes in infected wheat tissues was relatively low, they had different expression patterns during plant infection. In comparison with the others, Ps159 had a relatively high transcript level in infected wheat plants. Its expression was up-regulated from 2 to 6 dpi and then slightly decreased at 7 dpi. The transcript level of Ps85 and Ps28 increased at early infection stages but decreased after 5 dpi. The transcript level of Ps259 and Ps261 in wheat tissues was lower than that of the other ESTs examined. Those two genes may be specifically and highly expressed during urediniospore germination. These data suggest that a considerable reprogramming of gene expression occurs during urediniospore germination, early stages of plant infection, and the biotrophic growth in *P. striiformis *f. sp. *tritici*.

During incompatible interactions, Ps87 also displayed an expression pattern that decreased at early stages but slightly increased after 3 dpi (Fig. 4). The high transcript level of Ps87 in germinated urediniospores suggests that it may be involved in germ tube growth and appressorium differentiation. It is possible that Ps87 also plays a role in late stages of plant infection, probably for host-pathogen recognitions.

## Discussion

For plant pathogens with little or no history of genetic research, single-pass sequencing of random cDNA clones as in EST projects represents a relatively inexpensive and rapid procedure for finding novel genes and information about their expression. To date, extensive EST databases have been established for various plant pathogenic fungi, including *M. grisea *[43], *Phakopsora pachyrhizi *[52], and *F. oxysporum *[44]. For wheat rust fungi, only small EST libraries have been described for *P. triticina *[45] and *P. graminis *[12]. Recently, a full-length cDNA library has been constructed with RNA isolated from un-germinated urediniospores of a race PST-78 isolate of *P. striiformis *f. sp. *tritici *[46]. Among 196 random cDNA clones sequenced from this library, only 73 of them (37.2%) have homologs of known functions. Most of them are involved in various housekeeping functions. A few ESTs encoding hypothetical proteins have homologs in other plant pathogenic fungi [46]. Different from the cDNA library constructed in this study, the library constructed by Ling and colleagues may have high percentage of storage transcripts in un-germinated urediniospores.

Urediniospore germination represents an early stage of the interaction of *P. striiformis *f. sp. *tritici *with host plants. As an obligate biotroph, germ tubes of the wheat stripe rust fungus fail to progress beyond this stage of development *in vitro *[47,48]. In this study, we constructed and sequenced a cDNA library with RNA isolated from germinated urediniospores. Different from other filamentous fungi, a relatively high level of redundancy was found in this *P. striiformis *f. sp. *tritici *library. Among the 4798 ESTs generated in this study, 3995 of them could be assembled into contigs. A few contigs consisting of over 100 EST clones may represent genes that are highly expressed during urediniospore germination. When the uniseqs were queried against the nr protein database, about 76.2% of them had no significant homology with proteins of known functions, which may be related to the difficulty of functional characterization of genes in rust fungi. Among these contigs encoding proteins of known functions, 18 of them are ribosomal proteins. ESTs corresponding to genes encoding ribosomal proteins also are abundant in other fungal ESTs, such as those of *B. graminis *[49] and *M. grisea *[50].

The 1118 uniseqs identified in this study contained a wide range of genes involved in different cellular functions. The most abundant genes during urediniospore germination were those involved in metabolic activities as well as those responsible for protein biosynthesis, which accounted for 10.29% and 5.28% of the uniseqs, respectively. A few genes are involved in RNA synthesis, cell signal and communication, cell structure and growth, and cell/organism defense, indicating that active metabolism and protein synthesis is important for urediniospore germination and germ tube growth. Several ESTs, including Ps8, Ps28, and Ps228, shared similarity to differentiation-related protein Inf24 from *U. appendiculatus*, which is specifically expressed during urediniospore germination [51]. Ps88 has similarity to a chitin synthase from *P. graminis *f. sp. *tritici*. Ps55 and Ps1010 were homologous to a deacetylase and a cell wall organization and biogenesis-related protein from *C. neoformans*, respectively [52]. These three genes may be involved in the modification of cell wall during urediniospore germination. The contig Ps5712 encodes a putative calcium/calmodulin-dependent protein kinase. The calcium-signaling pathway has been implicated in regulating appressorium formation in *M. grisea *and other plant pathogens [53,54]. In the wheat stripe rust fungus, it may regulate germ tube emergence and infection structure differentiation.

Expression patterns of six selected uniseqs, Ps28, Ps85, Ps87, Ps159, Ps261, and Ps295, were examined by qRT-PCR analysis. The transcript level of these genes in germinated urediniospores was several-folds higher than in un-germinated urediniospores or infected wheat tissues, indicating that gene expression in the wheat stripe rust fungus changes dramatically during urediniospore germination and parasitic growth in wheat plants (Fig. 3; Fig. 4). These observations are consistent with what have been reported in *U. fabae *[16].

A number of genes that are important for plant infection have been identified in various phytopathogenic fungi [55]. Several of them are key components of conserved signaling pathways. In the ESTs generated in this study, we identified several genes involved in signal transduction, such as Ps259 and Ps261 that encode a G-alpha subunit and a Pmk1 homolog, respectively. Similarly, several signaling components are identified in *P. triticina *ESTs, including the Pmk1 homolog *PtMAPK1 *[17]. The *PtMAPK1 *gene has increased transcript levels during urediniospore germination and plant infection. When expressed in *U. maydis*, it can complement the defects of the *kpp2 *mutant in mating and plant infection [56].

Other putative fungal virulence or pathogenicity factors identified in this EST analysis included contigs Ps8, Ps28, Ps6, Ps159, and Ps87. Contigs Ps8 and Ps28 shared similarity to differentiation-related protein Inf24 from *U. appendiculatus*. In *U. appendiculatus*, microinjection of Inf24 antisense fragment into germinated urediniospores blocked its transcription and appressorium formation [19]. Injection with sense fragments has no effect on responses of germ tubes to the topographical stimuli and development of appressoria. Injection of antisense fragments into mature appressoria has no inhibitory effects on the development of subsequent infection structures. The Inf24 protein appears to play a critical role in the germ tube before the formation of appressoria. The presence of Inf24 homologs in this EST library of germinated urediniospores suggested that they may have similar functions in the wheat stripe rust fungus.

Ps6 and Ps159 have similarity to *GAS1 *and *GAS*2 of *M. grisea *[34], respectively. *GAS*1 and *GAS*2 are specifically expressed during appressorium formation and important for appressorial penetration in *M. grisea*. They are homologous to gEgh16 of *B. graminis *and members of a small protein family unique to filamentous fungi. In several fungal pathogens, gEgh16 homologs are expressed at early plant infection stages [23,34]. In the wheat stripe rust and leaf rust fungi [17], gEgh16 homologs may be involved in early stages of appressorium development. Although Ps159 was transcribed at a level higher than those of other ESTs in infected plant tissues, its transcript was much more abundant in germinated urediniospores (Fig. 3). After germinating for 10 h, the germ tube tips tend to swell in *P. striiformis *f. sp. *tritici *(Fig. 1). Genes involved in appressorium formation may be highly expressed at this stage.

When reliable transformation systems become available for *P. striiformis *f. sp. *tritici *in the future, it will be important to determine the functions of these putative pathogenicity factors identified in this EST library by generating gene knockout or silencing mutants. Some of these genes highly expressed in germinated urediniospores may play important roles in fungal-plant interactions during early infection stages in the wheat stripe rust fungus.

## Conclusion

A cDNA library was constructed from germinated urediniospores of *P. striiformis *f. sp. *tritici*. A total of 4798 ESTs were sequenced and assembled into 315 contigs and 803 singletons. About 62.8% of the resulting 1118 uniseqs had no significant homologs in GenBank. Among the uniseqs with assigned functions, over 70% of them were functionally related to primary metabolism and protein or RNA synthesis. The rest were associated with various cellular functions. Several of them were homologous to known fungal pathogenicity factors or effector proteins. The high transcript level of six selected ESTs in germinated urediniospores was confirmed by qRT-PCR. Genes identified in this study to be highly expressed in germinated urediniospores may be important for early infection processes in *P. striiformis *f. sp. *tritici*.

## Methods

### Strains and culture conditions

*P. striiformis *f. sp. *tritici *strain CY32 was inoculated and propagated on wheat cultivar Huixianhong as described previously [57]. Fresh urediniospores were harvested from infected wheat plants and resuspended in sterile distilled water (6 mg/200 ml). After incubating in a 50 × 125 cm dish at 9°C for 10 h, germinated urediniospores were collected with a spatula, frozen in liquid nitrogen, and stored at -80°C. For isolating RNA from infected plants, wheat leaves of susceptible cultivar Huixianhong and resistant cultivar Shuiyuan 11 were inoculated with CY32 urediniospores and harvested at 1, 2, 3, 4, 5, 6, and 7 days post inoculation (dpi).

### cDNA synthesis, library construction, and DNA sequencing

Total RNA was isolated from 100 mg of germinated urediniospores with the RNeasy Plant Mini RNA purification kit (QIAGEN, Germany) following the instruction provided by the manufacturer. The SMART^TM ^cDNA library construction kit (Clontech, USA) was used for cDNA synthesis and library construction. The MaxPlax^TM ^Lambda Packaging Extract (Epicentre, USA) was used for *in vitro *packaging and transfection of *Escherichia coli *strain XL1-Blue. The resulting directional library consisting of 6.05 × 10^5 ^primary clones was amplified and stored in 15% glycerol at -80°C. Randomly selected cDNA clones were sequenced with primer seq1 (5' CGACTCTAGACTCGAGCAAG 3') from the 5'-end with an ABI 3130-XL DNA sequencer.

### Sequence analysis and bioinformatics

Sequence reads longer than 100 base pairs were processed by cross_match [58] that allows the removal of contaminated sequences. Repeats and low complexity sequences were masked using RepeatMasker [59]. The resulting quality trimmed sequences were extracted and assembled with the CAP3 assembler, and viewed with Consed [60]. Statistics of the assemblies were generated by perl scripts using BioPerl modules.

For functional classification, the resulting unisequences were searched against the NCBI non-redundant protein database using the BLASTX program [61,62]. The unisequences with significant BLASTX matches were classified according to their likely cellular functions following the general categories outlined by the Gene Ontology Consortium [63]. InterProScan was used to search for protein domains in ESTs with no significant homologs in BLASTX searches.

### Isolating RNA from urediniospores, germinated urediniospores, and infected wheat leaves

Standard protocols [64] were used to extract total RNA from urediniospores, germinated urediniospores, and wheat leaves inoculated with the stripe rust fungus. Two micrograms of total RNA each was used for cDNA synthesis with the SuperScript First-strand Synthesis System (Invitrogen) following the instruction provided by the manufacture. The resulting 1^st^-strand cDNA products synthesized with RNA from urediniospores, germinated urediniospores, and infected wheat leaves were used as the templates for qRT-PCR assays.

### Quantitative real-time PCR analysis

Four sets of primers and fluorescently labeled TaqMan probes [see Additional file 5] were used to assay the transcript levels of ESTs Ps28, Ps85, Ps159, Ps259, Ps261, and Ps87. The expected sizes of resulting PCR products were 107, 65, 81, 73, 103, and 179 bp for Ps28, Ps85, Ps87, Ps159, Ps259, and Ps261, respectively. The fluorogenic probes were labeled at the 5'end with the fluorescent reporter dye FAM (6-carboxy-fluorescein) and modified at the 3'end with the quencher dye TAMRA (6-carboxy-tetramethylrhodamine). Transcript abundance was assessed with three independent biological replicates. Each real-time PCR mixture (25 μl) contained 12.5 μl premix (Premix Ex TaqTM, Takara), 7.5 μl distilled H_2_O, 1 μl 10 μM forward primer, 1 μl 10 μM reverse primer, 1 μl TaqMan probe, and 2 μl the cDNA template. The Smart CyclerSystem (Cepheid, USA) was used for PCR reactions that consisted of 10 s at 95°C (1 cycle) and 40 cycles of 95°C for 5 s and 60°C for 20 s. The transcript level of selected ESTs was calculated by the 2^-ΔΔCT ^method [65] with the actin gene of *P. striiformis *f. sp. *tritici *as the endogenous reference for normalization. Relative quantification of these ESTs was computed with their transcript levels in different stages in comparison to that in urediniospores.

## Authors' contributions

YZ constructed the cDNA library, participated in EST sequencing and sequence analysis, conducted qRT-PCR assays, and prepared the manuscript; ZQ, LX and XW contributed to EST sequencing and Blast searches. WZ participated in various experiments; BL and XX assisted in qRT-PCR assays; LH, JZ and QH coordinated the project; ZK conceived of this study, contributed to experimental designs, provided supervisions, and revised the manuscript; All authors read and approved the final manuscript.

## Supplementary Material

Additional file 1 The redundancy of *P. striiformis *f. sp *tritici *ESTs derived from the germinated urediniospore cDNA library. The number of contigs consisting of 1, 2, 3–5, 6–10, 11–35, and more than 36 ESTs was presented by the columns. Click here for file

Additional file 2 The most abundant ESTs identified in the *P. striiformis *f. sp. *tritici *germinated urediniospore library. These data provided represent the nine most abundant expressed ESTs. Click here for file

Additional file 3 Uniseqs with significant homology to genes from different organisms. These data provided display the number of uniseqs have homology to genes from diversity organisms. Click here for file

Additional file 4 Uniseqs with similarity (BLASTX, E-value < 10-5 or InterProScan, E-value < 10-5) to proteins in public databases were grouped into functional categories according to Gene Ontology. These data provided represent the original EST number and best hit. Click here for file

Additional file 5 Sequences of primers and probes used for qRT-PCR. The primer pairs, probes and reference sequences used for qRT-PCR were listed. Click here for file
