# Supplementary figures and images for: Stage-specific gene expression during urediniospore germination in Puccinia striiformis f. sp tritici
Source: BMC Genomics. 2008 May 1;9:203. doi: 10.1186/1471-2164-9-203 (PMC2386484; doi:10.1186/1471-2164-9-203)

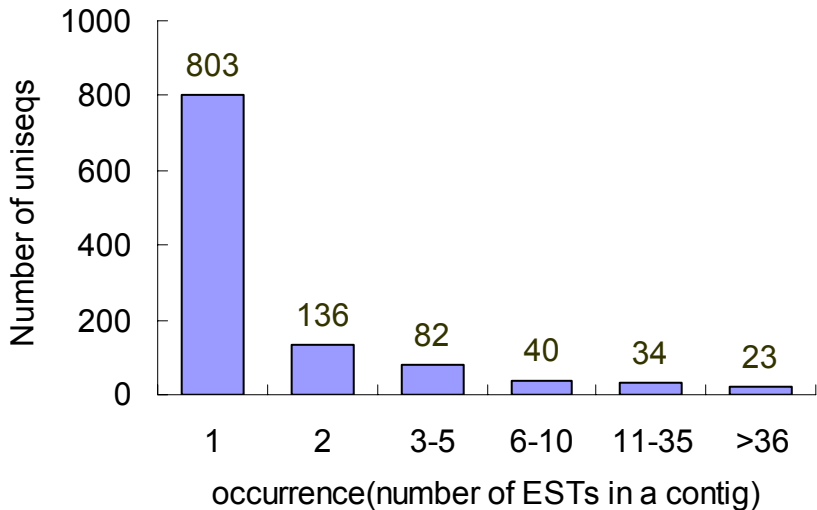

Supplement: Additional file 1 — The redundancy of P. striiformis f. sp tritici ESTs derived from the germinated urediniospore cDNA library. The number of contigs consisting of 1, 2, 3–5, 6–10, 11–35, and more than 36 ESTs was presented by the columns. [file 1471-2164-9-203-S1.pdf]
