# Supplementary material for: Stage-specific gene expression during urediniospore germination in Puccinia striiformis f. sp tritici
Source: BMC Genomics. 2008 May 1;9:203. doi: 10.1186/1471-2164-9-203 (PMC2386484; doi:10.1186/1471-2164-9-203)
Supplement: Additional file 2 — The most abundant ESTs identified in the P. striiformis f. sp. tritici germinated urediniospore library. These data provided represent the nine most abundant expressed ESTs. [file 1471-2164-9-203-S2.doc]

**Additional file 2:** The most abundant ESTs identified in the *P. striiformis* f. sp. *tritici* germinated urediniospore library

Contig No. of ESTsBest homolog in GenBankE-valuePs66101No hits found-Ps253110Putative secreted protein from *Ixodes scapularis* 9e-06Ps129121No hits found-Ps198122No hits found-Ps252127No hits found-Ps28128*Uromyces appendiculatus* differentiation-related protein Infp 3e-11Ps314154Predicted protein from *Kluyveromyces lactis* 2e-10Ps24182No hits found-Ps303608Predicted protein from *Saccharomyces cerevisiae*1e-10
