# Supplementary material for: Stage-specific gene expression during urediniospore germination in Puccinia striiformis f. sp tritici
Source: BMC Genomics. 2008 May 1;9:203. doi: 10.1186/1471-2164-9-203 (PMC2386484; doi:10.1186/1471-2164-9-203)
Supplement: Additional file 3 — Uniseqs with significant homology to genes from different organisms. These data provided display the number of uniseqs have homology to genes from diversity organisms. [file 1471-2164-9-203-S3.doc]

**Additional file 3:** Uniseqs with significant homology to genes from different organisms.

Filamentous fungiYeastBacteriaPlantanimalInsectNematodeNumber of uniseqs1691626304521% total uniseqs40.7239.041.457.2311.840.480.24
