# Supplementary material for: Stage-specific gene expression during urediniospore germination in Puccinia striiformis f. sp tritici
Source: BMC Genomics. 2008 May 1;9:203. doi: 10.1186/1471-2164-9-203 (PMC2386484; doi:10.1186/1471-2164-9-203)
Supplement: Additional file 4 — Uniseqs with similarity (BLASTX, E-value < 10-5 or InterProScan, E-value < 10-5) to proteins in public databases were grouped into functional categories according to Gene Ontology. These data provided represent the original EST number and best hit. [file 1471-2164-9-203-S4.doc]

**Additional file 4: Uniseqs displaying similarity (BLASTX, E value<10^-5^ or InterScan, E value<10^-5^) to proteins in public databases were grouped into functional categories according to Gene Ontology**

| Uniseqs | Length(bp) | Description | Species | E value | No.of clones | Organism |
| --- | --- | --- | --- | --- | --- | --- |
| 1 cell division and chromosome structure | | |  |  |  |  |
| Ps215 | 536 | Histone | *Cryptococcus neoformans* | 1.6E-17 | 2 | yeast |
| Ps_4020 | 464 | Eukaryotic DNA topoisomerase I | *Schizosaccharomyces pombe* | 6E-29 | 1 | yeast |
| Ps140 | 896 | Cyclin | *Cryptococcus neoformans* | 5E-16 | 10 | yeast |
| Ps_407 | 714 | cyclin-dependent protein kinase | *Schizosaccharomyces pombe* | 6E-24 | 1 | yeast |
| Ps57 | 1215 | nucleus protein, putative | *Cryptococcus neoformans* | 5E-26 | 20 | yeast |
| Ps94 | 541 | DNA polymerase processivity factor | *Cryptococcus neoformans* | 8E-46 | 2 | yeast |
| Ps_799 | 746 | meiotic recombination-related protein, putative | *Cryptococcus neoformans* | 1E-31 | 1 | yeast |
| Ps_553 | 752 | Thymidylate synthase | *Saccharomyces cerevisiae* | 2E-61 | 1 | yeast |
| Ps_1707 | 721 | Thymidylate synthase | *Saccharomyces cerevisiae* | 6E-74 | 1 | yeast |
| Ps_1972 | 681 | putative S-phase specific ribosomal protein cyc07 | *Lentinul aedodes* | 5E-80 | 1 | filamentous fungus |
| Ps_228 | 524 | CG6363-PA | *Drosophila melanogaster* | 3E-13 | 1 | animal |
| Ps_230 | 655 | hypothetical protein UM04018.1 | *Ustilago maydis* | 8E-22 | 1 | filamentous fungus |
| Ps_3505 | 467 | exodeoxyribonuclease III | *Bacillus thuringiensis* | 8E-10 | 1 | bacteria |
| Ps_4202 | 330 | AI449441 protein | *Mus musculus* | 4E-06 | 1 | animal |
| Ps_4444 | 626 | chromatin assembly factor 1 subunit C | *Aspergillus fumigatus* | 3E-42 | 1 | filamentous fungus |
| Ps_4489 | 558 | hypothetical protein DEHA0B05500g | *Debaryomyces hansenii* | 3E-07 | 1 | yeast |
|  |  |  |  |  |  |  |
| 2 Cell signal and communication | | |  |  |  |  |
| Ps_1728 | 260 | adenylate cyclase | *Magnaportha grisea* MAC1 | 3E-08 | 1 | filamentous fungus |
| Ps_446 | 770 | hypothetical protein UM05499.1 | *Ustilago maydis* | 3E-10 | 1 | filamentous fungus |
| Ps259 | 647 | G protein alpha subunit | *Ustilago maydis* *gpa1* | 2E-45 | 2 | filamentous fungus |
| Additional file 4 (*continued*) | | | | | | |
| Uniseqs | Length(bp) | Description | Species | E value | No.of clones | Organism |
| Ps_5159 | 458 | Calcium-binding EF-hand | *Cryptococcus neoformans* | 2E-63 | 1 | yeast |
| Ps306 | 612 | Protein kinase |  | NA | 2 |  |
| Ps261 | 776 | pathogenicity MAP kinase 1; Pmk1 | Magnaporthe grisea PMK1 | 2E-25 | 5 | filamentous fungus |
| Ps_5712 | 403 | calcium/calmodulin-dependent protein kinase | *Aspergillus fumigatus* | 6E-45 | 1 | filamentous fungus |
| Ps53 | 615 | Thiol peroxidase | *Saccharomyces cerevisiae* | 3E-55 | 2 | yeast |
| Ps116 | 789 | Protein kinase | *Ustilago maydis* | 1E-103 | 2 | filamentous fungus |
| Ps139 | 477 | glucose-repressible gene protein-related protein | *Aspergillusfumigatus* | 2E-14 | 24 | filamentous fungus |
| Ps250 | 428 | glucose-repressible protein grg-1 | *Neurospora crassa* | 1E-14 | 17 | filamentous fungus |
| Ps186 | 773 | Rickettsia 17 kDa surface antigen |  | 4E-07 | 13 |  |
| Ps_1090 | 734 | hypothetical protein UM01276.1 | *Ustilago maydis 521* | 2E-77 | 1 | filamentous fungus |
| Ps_5340 | 527 | G-protein beta subunit Bpp1 | *Ustilago maydis* | 2E-50 | 1 | filamentous fungus |
| Ps_356 | 717 | hypothetical protein UM03316.1 | *Ustilago maydis 521* | 1E-38 | 1 | filamentous fungus |
| Ps_612 | 668 | Cdc2 cyclin-dependent kinase, putative | *Cryptococcus neoformansvar* | 1E-58 | 1 | yeast |
|  |  |  |  |  |  |  |
| 3 Cell structure and growth | | |  |  |  |  |
| 3.1 Cytoskeletal | |  |  |  |  |  |
| Ps25 | 576 | actin | *Puccinia graminis* | 2E-97 | 3 | filamentous fungus |
| Ps296 | 525 | actin-like protein | *Homo sapiens* | 1E-16 | 4 | animal |
| Ps168 | 727 | hypothetical protein UM05158.1 | *Ustilago maydis* | 2E-09 | 2 | filamentous fungus |
| Ps189 | 815 | alpha1 tubulin | *Coprinopsis cinerea* | 1E-121 | 3 | filamentous fungus |
| Ps_1452 | 641 | actin lateral binding protein | *Cryptococcus neoformansvar* | 1E-34 | 1 | yeast |
|  |  |  |  |  |  |  |
| 3.2 growth sporulation | | |  |  |  |  |
| Additional file 4 (*continued*) | | | | | | |
| Uniseqs | Length(bp) | Description | Species | E value | No.of clones | Organism |
| Ps272 | 484 | nuclear condensin complex subunit Smc2 | *Aspergillus fumigatus* | 7E-63 | 2 | filamentous fungus |
| Ps64 | 690 | hypothetical protein UM02396.1 | *Ustilago maydis* | 1E-41 | 2 | filamentous fungus |
| Ps_878 | >614 | Pistil-specific extensin-like protein |  | 1.50E-05 | 1 |  |
| Ps288 | 499 | conidiation protein 6 (con-6) | *Neurospora crassa* | 6E-10 | 7 | filamentous fungus |
| Ps301 | 871 | conidiation protein 6 (con-6) | *Neurospora crassa* | 2E-10 | 20 | filamentous fungus |
| Ps_4207 | 701 | similar to dynein light chain-2 isoform 2 | *Canis familiaris* | 2E-32 | 1 | animal |
| Ps_944 | 625 | Cellulose-binding, family II, bacterial type:Fibronectin,  type III | *Acidothermus cellulolyticus* | 6E-07 | 1 | filamentous fungus |
| Ps8 | 967 | differentiation-related protein Infp | *Uromyces appendiculatus* | 8E-10 | 14 | filamentous fungus |
| Ps28 | 989 | differentiation-related protein Infp | *Uromyces appendiculatus* | 3E-11 | 128 | filamentous fungus |
| Ps38 | 626 | hypothetical protein FG01596.1 | *Gibberella zeae* | 6E-21 | 2 | filamentous fungus |
| Ps55 | 1254 | deacetylase | *Cryptococcus neoformans* | 2E-48 | 15 | yeast |
| Ps88 | 775 | hypothetical class II chitin synthase | *Puccinia graminis f. sp.tritici* | 2E-79 | 2 | filamentous fungus |
| Ps122 | 1052 | rust transferred protein | *Uromyces striatus* | 7E-26 | 7 | filamentous fungus |
| Ps278 | 1081 | hypothetical protein UM02019.1 | *Ustilago maydis* | 8E-27 | 2 | filamentous fungus |
| Ps_1010 | 307 | cell wall organization and biogenesis-related protein, putative | *Cryptococcus neoformans* | 3E-16 | 1 | yeast |
| Ps228 | 1016 | differentiation-related protein Infp | *Uromyces appendiculatus* | 3E-04 | 141 | filamentous fungus |
| Ps85 | 722 | mycelial surface antigen precursor | *Candida albicans* | 1E-05 | 63 | yeast |
|  |  |  |  |  |  |  |
| 4 Metablism | |  |  |  |  |  |
| 4.1 Amino Acid | |  |  |  |  |  |
| Ps124 | 945 | Peptidase C19, ubiquitin carboxyl-terminal hydrolase 2 |  | 2.3E-10 | 4 |  |
| Additional file 4 (*continued*) | | | | | | |
| Uniseqs | Length(bp) | Description | Species | E value | No.of clones | Organism |
| Ps176 | 692 | Peptidase C15, pyroglutamyl peptidase I |  | 1.70E-05 | 2 |  |
| Ps34 | 642 | novel protein similar to bacterial GDP-mannose  4,6-dehydratases | *Danio rerio* | 1E-63 | 3 | animal |
| Ps100 | 681 | unnamed protein product | *Aspergillus oryzae* | 2E-13 | 2 | filamentous fungus |
| Ps312 | 723 | hypothetical protein UM04562.1 | *Ustilago maydis* | 3E-85 | 5 | filamentous fungus |
| Ps_2229 | 276 | Arginine biosynthesis-related protein | *Cryptococcus neoformans* | 4E-07 | 1 | yeast |
| Ps_3340 | 376 | ubiquitin carboxyl-terminal hydrolase 5, putative | *Cryptococcus neoformans* | 9.2E-22 | 1 | yeast |
| Ps_1748 | 419 | hypothetical protein UM06448.1 | *Ustilago maydis* | 2E-25 | 1 | filamentous fungus |
| Ps209 | 805 | adenosylmethionine decarboxylase, putative | *Cryptococcus neoformansvar* | 4E-40 | 6 | yeast |
| Ps96 | 1548 | hypothetical protein CHGG_03456 | *Chaetomium globosum* | 4E-39 | 11 | filamentous fungus |
| Ps_6010 | 628 | hypothetical protein UM06017.1 | *Ustilago maydis* | 2E-47 | 1 | filamentous fungus |
| Ps_167 | 662 | Hydantoinase B/oxoprolinase | *Ustilago maydis* | 2.3E-41 | 1 | filamentous fungus |
| Ps_5756 | 637 | phytase, putative | *Cryptococcus neoformans* | 4E-41 | 1 | yeast |
| Ps45 | 581 | tetrahydrofolylpolyglutamate synthase, putative | *Cryptococcusneoformans* | 3E-34 | 2 | yeast |
| Ps113 | 715 | hypothetical protein | *Neurospora crassa* | 1E-21 | 2 | filamentous fungus |
| Ps263 | 708 | hypothetical protein UM04262.1 | *Ustilago maydis* | 2E-73 | 2 | filamentous fungus |
| Ps_3170 | 600 | hypothetical protein UM02848.1 | *Ustilago maydis* | 2E-36 | 1 | filamentous fungus |
| Ps_5173 | 661 | hypothetical protein UM05122.1 | *Ustilago maydis* | 6E-22 | 1 | filamentous fungus |
| Ps_721 | 605 | fuSed3 protease | *Aspergillus fumigatus* | 4E-40 | 1 | filamentous fungus |
| Ps_923 | 497 | glycine hydroxymethyltransferase | *Cryptococcus neoformansvar* | 7E-42 | 1 | yeast |
|  |  |  |  |  |  |  |
| 4.2 TCA cycle/Oxidative Phosphorylation/Electron Transport | | |  |  |  |  |
| Ps154 | 705 | hypothetical protein UM02899.1 | *Ustilago maydis* | 8E-60 | 3 | filamentous fungus |
| Additional file 4 (*continued*) | | | | | | |
| Uniseqs | Length(bp) | Description | Species | E value | No.of clones | Organism |
| Ps260 | 721 | NADH ubiquinone oxidoreductase, 20 kDa subunit | *Cryptococcus neoformans* | 1E-27 | 2 | yeast |
| Ps276 | 848 | Cytochrome oxidase c, subunit VIb | *Cryptococcus neoformans* | 9E-25 | 2 | yeast |
| Ps105 | 670 | Cytochrome c | *Alligator mississippiensis* | 2E-38 | 6 | animal |
| Ps_599 | 626 | Cytochrome-c oxidase chain VI precursor | *Cryptococcus neoformansvar* | 1.3E-36 | 1 | yeast |
| Ps177 | 680 | NADH-ubiquinone oxidoreductase ASHI subunit | *Cryptococcus neoformansvar* | 4E-17 | 3 | yeast |
| Ps41 |  | Ubiquinol-cytochrome C reductase, UQCRXQCR9 like |  | 1.8E-10 | 7 |  |
| Ps268 |  | Aldehyde dehydrogenase |  | NA | 5 |  |
| Ps39 | 574 | Cytochrome c oxidase, subunit VIa | *Paracoccidioides brasiliensis* | 3.7E-23 | 4 | filamentous fungus |
| Ps200 | 626 | hypothetical protein UM00092.1 | *Ustilago maydis* | 9E-25 | 3 | filamentous fungus |
| Ps_5877 | 576 | hypothetical protein UM01881.1 | *Ustilago maydis* | 4E-23 | 1 | filamentous fungus |
| Ps149 | 624 | Electron transfer flavoprotein beta-subunit | *Neurospora crassa* | 2E-22 | 3 | filamentous fungus |
| Ps_2071 | 472 | SPAC23C4.11 | *Schizosaccharomyces pombe* | 1E-09 | 1 | yeast |
| Ps_4985 | 541 | ard1 family protein, putative | *Cryptococcus neoformans* | 3E-55 | 1 | yeast |
| Ps_5642 | 760 | glucosamine 6-phosphate N-acetyltransferase | *Cryptococcus neoformans* | 6E-24 | 1 | yeast |
| Ps273 | 713 | pyrroline-5-carboxylate reductase, putative | *Cryptococcus neoformans* | 6E-34 | 6 | yeast |
| Ps_2154 | 625 | enoyl-CoA hydratase/isomerase family protein | *Aspergillus fumigatus* | 3E-26 | 1 | filamentous fungus |
| Ps_2127 | 584 | fumarate reductase (NADH) | *Cryptococcus neoformans* | 2E-68 | 1 | yeast |
| Ps_4684 | 600 | hypothetical protein UM05130.1 | *Ustilago maydis* | 2E-28 | 1 | filamentous fungus |
| Ps_5186 | 697 | probable 3-isopropylmalate dehydratase | *Neurospora crassa* | 2E-69 | 1 | filamentous fungus |
| Ps_5400 | >600 | 2Fe-2S ferredoxin, iron-sulfur binding site |  | NA | 1 |  |
| Ps206 | 700 | hypothetical protein UM00778.1 | *Ustilago maydis* | 6E-18 | 6 | filamentous fungus |
| Ps270 | 649 | hypothetical protein UM04467.1 | *Ustilago maydis* | 2E-42 | 3 | filamentous fungus |
| Ps92 | 498 | GTP cyclohydrolase II | *Aspergillus fumigatus* | 2E-11 | 2 | filamentous fungus |
| Additional file 4 (*continued*) | | | | | | |
| Uniseqs | Length(bp) | Description | Species | E value | No.of clones | Organism |
| Ps_788 | 755 | hypothetical protein CHGG_04378 | *Chaetomium globosum* | 1E-11 | 1 | filamentous fungus |
| Ps_1007 | 377 | acetohydroxyacid reductoisomerase | *Filobasidiella neoformans* | 1E-06 | 1 | filamentous fungus |
| Ps_1894 | 749 | hypothetical protein UM01213.1 | *Ustilago maydis* | 1E-26 | 1 | filamentous fungus |
| Ps_2483 | 658 | ketol-acid reductoisomerase, putative | *Cryptococcus neoformans* | 3E-99 | 1 | yeast |
| Ps_3717 | 695 | mitochondrion protein, putative | *Cryptococcus neoformans* | 7E-11 | 1 | yeast |
|  |  |  |  |  |  |  |
| 4.3 Lipid/Fatty Acid | | |  |  |  |  |
| Ps145 | 904 | SPAC26A3.11 | *Schizosaccharomyces pombe* | 2E-73 | 2 | yeast |
| Ps_429 | 754 | Acyl-CoA dehydrogenase | *Cryptococcus neoformans* | 2E-72 | 1 | yeast |
| Ps_4939 | 643 | hypothetical protein UM02063.1 | *Ustilago maydis* | 9E-33 | 1 | filamentous fungus |
| Ps_5644 | 684 | hypothetical protein Afu3g01920 | *Aspergillus fumigatus* | 4E-51 | 1 | filamentous fungus |
| Ps_1132 | 675 | glycerol-1-phosphatase, putative | *Cryptococcus neoformans* | 1E-19 | 1 | yeast |
| Ps_1661 | 615 | hypothetical protein UM05286.1 | *Ustilago maydis* | 1E-32 | 1 | filamentous fungus |
| Ps190 | 605 | fatty acid beta-oxidation-related protein, putative | *Cryptococcus neoformans* | 2E-44 | 2 | yeast |
| Ps_600 | 678 | acyl-protein thioesterase-1, putative | *Cryptococcus neoformans* | 1E-40 | 1 | yeast |
| Ps_594 | 646 | hypothetical protein CaO19_9566 | *Candida albicans* | 1E-20 | 1 | yeast |
| Ps_1192 | 671 | hypothetical protein FG02073.1 | *Gibberella zeae* | 5E-08 | 1 | filamentous fungus |
| Ps_1405 | 236 | hypothetical protein | *Yarrowia lipolytica* | 6E-07 | 1 | plant |
| Ps_869 | 609 | phospholipid:diacylglycerol acyltransferase, putative | *Cryptococcus neoformans* | 4E-18 | 1 | yeast |
| Ps_56 | 671 | Phospholipase A2, active site |  | NA | 1 |  |
| Ps_4593 | 457 | Protein involved in sphingolipid biosynthesis;  type II membraneprotein with similarity to Kre6p; Skn1p | *Saccharomycescerevisiae* | 4E-16 | 1 | yeast |
|  |  |  |  |  |  |  |
| Additional file 4 (*continued*) | | | | | | |
| Uniseqs | Length(bp) | Description | Species | E value | No.of clones | Organism |
| 4.4 Carbon Metabolism | | |  |  |  |  |
| Ps_1225 | 487 | saccharopine dehydrogenase | *Aspergillus fumigatus* | 3E-42 | 1 | filamentous fungus |
| Ps10 | 1241 | carbohydrate deacetylase | *Cryptococcus gattii* | 3E-47 | 22 | yeast |
| Ps68 | 1090 | carbohydrate deacetylase | *Cryptococcus gattii* | 1E-52 | 26 | yeast |
| Ps153 | 1308 | rAsp f 9 | *Aspergillus fumigatus* | 9E-16 | 7 | filamentous fungus |
| Ps_2128 | 354 | carbohydrate deacetylase | *Cryptococcus gattii* | 6E-13 | 1 | yeast |
| Ps_3585 | 421 | Glucose/ribitol dehydrogenase | *Cryptococcus neoformans* | 5E-10 | 1 | yeast |
| Ps_1947 | >689 | Carbohydrate-binding family V/XII |  | 1.3E-06 | 1 |  |
| Ps_5980 | 5980 | Transaldolase | *Candida albicans* | 3E-66 | 1 | yeast |
| Ps_2438 | 526 | dienelactone hydrolase family protein | *Aspergillus fumigatus* | 4E-15 | 1 | filamentous fungus |
| Ps86 | 587 | glucan 1,3 beta-glucosidase protein putative | *Cryptococcus neoformans* | 1E-12 | 3 | yeast |
| Ps121 | 532 | beta glucosidase precursor | *Uromyces viciae-fabae* | 5E-29 | 2 | filamentous fungus |
| Ps_148 | 861 | alpha,alpha-trehalose-phosphate synthase (UDP-forming), putative | *Cryptococcus neoformans* | 8E-51 | 1 | yeast |
| Ps_622 | 602 | exo-beta-1,3-glucanase | *Cryptococcus neoformans* | 1E-48 | 1 | yeast |
| Ps266 | 708 | LARGE (mouse dystroglycan glycosylation) homolog family member(lge-1) | *Caenorhabditis elegans* | 1E-08 | 1 | nematode |
| Ps_968 | 598 | triose phosphate/3-phosphoglycerate/phosphate  translocator,putative | *Cryptococcus neoformans* | 2E-16 | 1 | yeast |
|  |  |  |  |  |  |  |
| 4.5 Plant Cell Degradation | | |  |  |  |  |
| Ps74 | 1154 | serine esterase, cutinase family | *Ustilago maydis* | 2E-12 | 76 | filamentous fungus |
| Ps289 | 585 | cellulase | *Filobasidiella neoformans* | 1E-23 | 3 | filamentous fungus |
| Additional file 4 (*continued*) | | | | | | |
| Uniseqs | Length(bp) | Description | Species | E value | No.of clones | Organism |
|  |  |  |  |  |  |  |
| 4.6 transport | |  |  |  |  |  |
| Ps287 | 590 | P-type cation-transporting ATPase | *Blastocladiella emersonii* | 5E-06 | 2 | filamentous fungus |
| Ps_5240 | 744 | conserved hypothetical protein | *Cryptococcus neoformans* | 1E-46 | 1 | yeast |
| Ps29 | 633 | H+-transporting two-sector ATPase, A subunit | *Ustilago maydis* | 3E-29 | 2 | filamentous fungus |
| Ps185 | 662 | vacuolar ATP synthase 16 kDa proteolipid subunit | *Ustilago maydis* | 2E-63 | 4 | filamentous fungus |
| Ps271 | 721 | H+-transporting two-sector ATPase, delta/epsilon subunit | *Cryptococcus neoformans* | 1E-44 | 3 | yeast |
| Ps293 | 548 | VA0E_CRYNE Vacuolar ATP synthase subunit e  (V-ATPase e subunit) | *Chaetomium globosum* | 1E-08 | 2 | filamentous fungus |
| Ps_1638 | 642 | H+-transporting two-sector ATPase, D subunit | *Candida albicans* | 2E-39 | 1 | yeast |
| Ps229 | 671 | H+-transporting two-sector ATPase, alpha/beta subunit,  central region |  | NA | 4 |  |
| Ps_3048 | 714 | calcium-transporting ATPase | *Pichia pastoris* | 7E-14 | 1 | yeast |
| Ps_2082 | 674 | Mitochondrial import inner membrane translocase, subunit Tim17/22 | *Cryptococcus neoformans* | 9E-45 | 1 | yeast |
| Ps300 | 1112 | ABC transporter(ATP-binding cassette transporter) related | *Cryptococcus neoformans* | 3E-92 | 3 | yeast |
| Ps_5709 | 352 | related to ATP-binding cassette transporter protein | *Magnaporthe grisea* | 3E-8 | 1 | filamentous fungus |
| Ps_609 | 666 | Mitochondrial substrate carrier | *Yarrowia lipolytica* | 9E-10 | 1 | plant |
| Ps_1057 | 648 | Mitochondrial substrate carrier | *Cryptococcus neoformans* | 2E-25 | 1 | yeast |
| Ps_5513 | 628 | Mitochondrial substrate carrier | *Cryptococcus neoformans* | 3E-34 | 1 | yeast |
| Ps205 | 736 | Transthyretin | *Cryptococcus neoformans* | 7E-28 | 3 | yeast |
| Additional file 4 (*continued*) | | | | | | |
| Uniseqs | Length(bp) | Description | Species | E value | No.of clones | Organism |
| Ps_1094 | >704 | inorganic phosphate transporter | *Schizosaccharomyces pombe* | 9E-19 | 1 | yeast |
| Ps_1168 | 337 | Phosphotransferase system, HPr histidine phosphorylation site |  | NA | 1 |  |
| Ps101 | 668 | amino acid transporter | *Uromyces fabae* | 3E-33 | 2 | filamentous fungus |
| Ps_2461 | 568 | vesicle-mediated transport-related protein, putative | *Cryptococcus neoformans* | 7E-68 | 1 | yeast |
| Ps_4432 | 716 | ER to Golgi transport-related protein, putative | *Cryptococcus neoformans* | 8E-39 | 1 | yeast |
| Ps_288 | 582 | hypothetical protein UM03530.1 | *Ustilago maydis* | 1E-17 | 1 | filamentous fungus |
| Ps_1976 | 734 | hypothetical protein UM00679.1 | *Ustilago maydis* | 3E-17 | 1 | filamentous fungus |
| Ps67 | 910 | hypothetical protein UM02437.1 | *Ustilago maydis* | 4E-27 | 6 | filamentous fungus |
| Ps_3592 | 725 | 28 kda golgi snare protein, putative | *Cryptococcus neoformans* | 6E-10 | 1 | yeast |
|  |  |  |  |  |  |  |
| 4.7 Other metabolism | | |  |  |  |  |
| Ps_4405 | 609 | Vitamin B6 biosynthesis protein | *Cryptococcus neoformans* | 2E-35 | 1 | yeast |
| Ps_479 | 694 | NADP-dependent alcohol dehydrogenase | *Aspergillus fumigatus* | 1E-42 | 1 | filamentous fungus |
| Ps128 | 564 | Alcohol dehydrogenase superfamily, zinc-containing | *Aspergillus oryzae* | 3E-21 | 3 | filamentous fungus |
| Ps_224 | 756 | thioltransferase; glutaredoxin | *Schizosaccharomyces pombe* | 2E-06 | 1 | yeast |
| Ps_4799 | 548 | Thioredoxin-related | *Aspergillus fumigatus* | 4E-12 | 1 | filamentous fungus |
| Ps_5912 | 790 | Pyridoxamine 5'-phosphate oxidase | *Ustilago maydis* | 3E-57 | 1 | filamentous fungus |
| Ps89 | 556 | thioredoxin-disulfide reductase | *Ustilago maydis* | 3E-47 | 3 | filamentous fungus |
| Ps262 | 515 | glyoxal oxidase precursor, putative | *Cryptococcus neoformans* | 9E-12 | 2 | yeast |
| Ps_5559 | 405 | hypothetical protein UM00854.1 | *Ustilago maydis* | 4E-27 | 1 | filamentous fungus |
| Ps_108 | 785 | conserved hypothetical protein | *Cryptococcus neoformans* | 3E-34 | 1 | yeast |
|  |  |  |  |  |  |  |
| 5 Cell/Organism defense | | | | | | |
| Additional file 4 (*continued*) | | | | | | |
| Uniseqs | Length(bp) | Description | Species | E value | No.of clones | Organism |
| 5.1 detoxification | | |  |  |  |  |
| Ps238 | 1060 | secreted Cu/Zn superoxide dismutase similarity to SOD1 | *Candida albicans* | 1E-13 | 9 | yeast |
| Ps_4933 | 676 | related to cytosolic Cu/Zn superoxidedismutase [imported] | *Neurospora crassa* | 1E-13 | 1 | filamentous fungus |
| Ps232 | 673 | copper-induced metallothionein | *Tetrahymena pigmentosa* | 1E-05 | 62 | animal |
|  |  |  |  |  |  |  |
| 5.2 DNA repair | |  |  |  |  |  |
| Ps_4235 | 646 | DNA mismatch repair protein MutS, C-terminal | *Ustilago maydis* | 5E-60 | 1 | filamentous fungus |
| Ps_2259 | 460 | DNA/RNA non-specific endonuclease | *Neurospora crassa* | 4E-42 | 1 | filamentous fungus |
|  |  |  |  |  |  |  |
| 5.3 stress response | | |  |  |  |  |
| Ps_2041 | 645 | Catalase |  | 4.8E-05 | 1 |  |
| Ps9 | 820 | Blue (type 1) copper domain |  | NA | 4 |  |
| Ps83 | 719 | putative senescence-associated protein | *Pisum sativum* | 7E-48 | 5 | plant |
| Ps290 | 1334 | related to stress response protein rds1p | *Neurospora crassa* | 3E-45 | 23 | filamentous fungus |
|  |  |  |  |  |  |  |
| 6 Protein biosythesis | | |  |  |  |  |
| 6.1 robosomal protein | | |  |  |  |  |
| Ps131 | 680 | 60S ribosomal protein L8 | *Schizosaccharomyces pombe* | 5E-96 | 2 | yeast |
| Ps151 | 749 | Ribosomal protein S24e | *Ustilago maydis* | 3E-42 | 6 | filamentous fungus |
| Ps164 | 470 | putative cytosolic ribosomal protein L15 | *Candida albicans* | 3E-69 | 2 | filamentous fungus |
| Ps172 | 571 | Ribosomal protein L14b/L23e | *Ustilago maydis* | 6E-40 | 2 | filamentous fungus |
| Ps227 | 617 | ribosomal protein S11, putative | *Cryptococcus neoformans* | 3E-65 | 2 | yeast |
| Additional file 4 (*continued*) | | | | | | |
| Uniseqs | Length(bp) | Description | Species | E value | No.of clones | Organism |
| Ps_1130 | 711 | 40s ribosomal protein s5-1 | *Cryptococcus neoformans* | 3E-86 | 1 | yeast |
| Ps_1969 | 567 | hypothetical protein UM02409.1 | *Ustilago maydis* | 9E-54 | 1 | filamentous fungus |
| Ps_2103 | 682 | related to ribosomal protein L30 | *Neurospora crassa* | 8E-09 | 1 | filamentous fungus |
| Ps_3534 | 559 | Ribosomal protein L33 | *Cryptococcus neoformans* | 2E-10 | 1 | yeast |
| Ps_3802 | 529 | 40s ribosomal protein s15 | *Cryptococcus neoformans* | 3E-41 | 1 | yeast |
| Ps_2886 | 526 | putative U3 snoRNP protein | *Candida albicans* | 8E-43 | 1 | yeast |
| Ps_3183 | 503 | PRCDNA35, putative | *Cryptococcus neoformans* | 1E-18 | 1 | yeast |
| Ps_5046 | 668 | hypothetical protein UM00408.1 | *Ustilago maydis* | 4E-38 | 1 | filamentous fungus |
| Ps_5202 | 656 | Ribosomal protein L6 | *Ustilago maydis* | 2E-63 | 1 | filamentous fungus |
| Ps_947 | 606 | structural constituent of ribosome | *Cryptococcus neoformans* | 2.5E-48 | 1 | yeast |
| Ps282 | 754 | Ribosomal protein L5 |  | NA | 2 |  |
| Ps_2478 | 289 | Ribosomal protein S19e |  | 3.4E-09 | 1 |  |
| Ps291 | 692 | ribosomal protein S18 | *Cryptococcus neoformans* | 1E-56 | 4 | yeast |
| Ps_901 | 501 | MIS3_SCHPO Ribosomal RNA assembly protein mis3 | *Schizosaccharomyces pombe* | 8E-55 | 1 | yeast |
| Ps302 | 419 | unnamed protein product | *Kluyveromyces lactis* | 6E-10 | 18 | yeast |
| Ps303 | 778 | unnamed protein product | *Kluyveromyces lactis* | 1E-10 | 608 | yeast |
| Ps_2696 | 496 | ribosomal protein S12, putative | *Cryptococcus neoformans* | 7E-16 | 1 | yeast |
| Ps_2788 | 363 | hypothetical protein AN3444.2 | *Aspergillus nidulans* | 4E-40 | 1 | filamentous fungus |
| Ps314 | 402 | unnamed protein product | *Kluyveromyces lactis* | 2E-10 | 154 | yeast |
| Ps315 | 405 | Tar1p | *Saccharomyces cerevisiae* | 3E-08 | 39 | yeast |
| Ps_5293 | 367 | unnamed protein product | *Kluyveromyces lactis* | 2E-18 | 1 | yeast |
| Ps_28 | 321 | unnamed protein product | *Kluyveromyces lactis* | 2E-18 | 1 | yeast |
| Ps249 | 673 | 40s ribosomal protein | *Aspergillus fumigatus* | 3E-39 | 3 | filamentous fungus |
| Additional file 4 (*continued*) | | | | | | |
| Uniseqs | Length(bp) | Description | Species | E value | No.of clones | Organism |
| Ps_958 | 175 | TAR1_YEAST Protein TAR | *Saccharomyces cerevisiae* | 5E-07 | 1 | yeast |
| Ps_2490 | 170 | TAR1_YEAST Protein TAR1 | *Saccharomyces cerevisiae* | 1E-07 | 1 | yeast |
| Ps_3633 | 165 | TAR1_YEAST Protein TAR1 | *Saccharomyces cerevisiae* | 1E-07 | 1 | yeast |
|  |  |  |  |  |  |  |
| 6.2 translation factors | | |  |  |  |  |
| Ps264 | 735 | hypothetical protein UM02665.1 | *Ustilago maydis* | 2E-28 | 4 | filamentous fungus |
| Ps_6038 | 678 | ADP-ribosylation factor-like protein | *Bombyx mori* | 5E-62 | 1 | animal |
|  |  |  |  |  |  |  |
| 6.3 tRNA Synthesis | | |  |  |  |  |
| Ps_417 | 724 | unnamed protein product | *Tetraodon nigroviridis* | 2E-37 | 1 | animal |
| Ps_4054 | 569 | pseudouridine synthase, putative | *Cryptococcus neoformans* | 7E-25 | 1 | yeast |
| Ps_1718 | 656 | tRNA-intron endonuclease, putative | *Cryptococcus neoformans* | 4E-12 | 1 | yeast |
|  |  |  |  |  |  |  |
| 6.4 protein turnover | | |  |  |  |  |
| Ps_5579 | 793 | proteasome subunit, beta type, 7 | *Cryptococcus neoformansvar* | 2E-69 | 1 | yeast |
| Ps_1656 | 576 | hypothetical protein UM02340.1 | *Ustilago maydis* | 2E-40 | 1 | filamentous fungus |
| Ps_1722 | 329 | ubiquitin-ribosomal protein fusion S27a | *Candida albicans* | 6E-27 | 1 | yeast |
| Ps184 | 710 | endopeptidase, putative | *Cryptococcus neoformans* | 1E-52 | 2 | yeast |
| Ps_4135 | 661 | E2 ubiquitin-conjugating enzyme, putative | *Cryptococcus neoformansvar* | 2E-71 | 1 | yeast |
| Ps93 | 475 | hypothetical protein AN6179.2 | *Aspergillus nidulans* | 3E-26 | 2 | filamentous fungus |
| Ps119 | 417 | ubiquitin-protein ligase, putative | *Cryptococcus neoformans* | 1E-06 | 2 | yeast |
| Ps305 | 484 | ubiquitin-carboxy extension protein fusion, putative | *Cryptococcus neoformans* | 2E-62 | 2 | yeast |
| Ps_3576 | 369 | ubiquitin-protein ligase, putative | *Cryptococcus neoformans* | 3E-40 | 1 | yeast |
| Additional file 4 (*continued*) | | | | | | |
| Uniseqs | Length(bp) | Description | Species | E value | No.of clones | Organism |
| Ps_3375 | 643 | hypothetical protein CNBA4030 | *Cryptococcus neoformans* | 3E-34 | 1 | yeast |
|  |  |  |  |  |  |  |
| 6.5 post-translation modification/trafficking | | |  |  |  |  |
| Ps_1137 | 588 | Microsomal signal peptidase 12 kDa subunit |  | 3.2E-23 | 1 |  |
| Ps_114 | 692 | Peptidase | *Cryptococcus neoformans* | 1E-64 | 1 | yeast |
| Ps112 | 652 | hypothetical protein FG01697.1 | *Gibberella zeae* | 4E-22 | 2 | filamentous fungus |
| Ps_294 | 637 | Peptidase M14, carboxypeptidase A |  | NA | 1 |  |
| Ps_2211 | 523 | Peptidase M20 | *Ustilago maydis* | 1E-43 | 1 | filamentous fungus |
| Ps_5791 | 631 | prolidase, putative | *Cryptococcus neoformans* | 1E-12 | 1 | yeast |
| Ps_2804 | 607 | peptide transporter MTD1 | *Schizophyllum commune* | 5E-28 | 1 | yeast |
| Ps21 | 601 | Cytochrome c oxidase assembly protein | *Ustilago maydis* | 9E-42 | 2 | filamentous fungus |
| Ps_1386 | 159 | F-actin capping protein, beta subunit | *Caenorhabditis remanei* | 3E-15 | 1 | yeast |
| Ps_6045 | 770 | GTPase | *Cryptococcus neoformans* | 2E-53 | 1 | yeast |
| Ps283 | 776 | putative polyubiquitin | *Arabidopsis thaliana* | 9E-95 | 7 | plant |
| Ps297 | 1030 | vps28 protein, putative | *Cryptococcus neoformans* | 7E-52 | 3 | yeast |
| Ps_326 | 771 | hypothetical protein UM00711.1 | *Ustilago maydis* | 1E-32 | 1 | filamentous fungus |
| Ps_87 | 621 | trafficking-related protein, putative | *Cryptococcus neoformans* | 2E-29 | 1 | yeast |
|  |  |  |  |  |  |  |
| 7 RNA synthesis | |  |  |  |  |  |
| 7.1 RNA polymerases | | |  |  |  |  |
| Ps_1716 | 596 | RNA polymerase | *Saccharomyces cerevisiae* | 8E-33 | 1 | yeast |
| Ps_231 | 618 | DNA-directed RNA polymerase | *Cryptococcus neoformans* | 8E-28 | 1 | yeast |
| Ps_361 | 355 | DNA-dependent RNA polymerase II RPB140, putative | *Cryptococcus neoformans* | 1E-06 | 1 | yeast |
| Additional file 4 (*continued*) | | | | | | |
| Uniseqs | Length(bp) | Description | Species | E value | No.of clones | Organism |
|  |  |  |  |  |  |  |
| 7.2 transcription regulation | | |  |  |  |  |
| Ps75 | 986 | SPBC1198.13c | *Schizosaccharomyces pombe* | 1E-11 | 3 | yeast |
| Ps_3450 | 729 | Transcription factor TFIIB related |  | 3.1E-09 | 1 |  |
| Ps_3412 | >653 | Basic-leucine zipper (bZIP) transcription factor |  | NA | 1 |  |
| Ps_5638 | 658 | Transcriptional coactivator | *Saccharomyces cerevisiae* | 3E-30 | 1 | yeast |
| Ps299 | 613 | Transcription elongation factor that contains a  conserved zincfinger domain | *Saccharomyces cerevisiae* | 1E-08 | 2 | yeast |
| Ps_3549 | 640 | transcriptional elongation regulator | *Cryptococcus neoformans* | 1E-19 | 1 | yeast |
| Ps_4019 | 563 | hypothetical protein | *Neurospora crassa* | 1E-25 | 1 | filamentous fungus |
| Ps_4291 | 762 | transcription initiation factor TFIIE, subunit beta | *Aspergillus fumigatus* | 5E-08 | 1 | filamentous fungus |
| Ps_4213 | 614 | hypothetical protein UM00173.1 | *Ustilago maydis* | 6E-13 | 1 | filamentous fungus |
| Ps_5699 | 446 | Helix-turn-helix, Fis-type |  | NA | 1 |  |
|  |  |  |  |  |  |  |
| 7.3 RNA procesing | |  |  |  |  |  |
| Ps201 | 445 | thioredoxin-like 4A | *Xenopus tropicalis* | 2E-49 | 2 | animal |
| Ps_5486 | 655 | hypothetical protein UM04446.1 | *Ustilago maydis* | 4E-19 | 1 | filamentous fungus |
| Ps62 | 499 | small nuclear ribonucleoprotein E | *Cryptococcus neoformans* | 6E-21 | 2 | yeast |
| Ps248 | 584 | pre-mRNA splicing factor, putative | *Cryptococcus neoformans* | 3E-45 | 3 | yeast |
| Ps_193 | 580 | Like-Sm ribonucleoprotein | *Cryptococcus neoformans* | 1E-31 | 1 | yeast |
| Ps_2116 | 743 | ZNF24 protein | *Homo sapiens* | 9E-12 | 1 | human |
| Ps71 | 692 | Zinc finger, C2H2-type |  | NA | 9 |  |
| Ps12 | 767 | hypothetical protein Lgas02000349 | *Lactobacillus gasseri* | 6E-09 | 3 | bacteria |
| Additional file 4 (*continued*) | | | | | | |
| Uniseqs | Length(bp) | Description | Species | E value | No.of clones | Organism |
| Ps183 | 932 | RNase E: endoribonuclease for rRNA processing and  mRNA degradation | *Pseudoalteromonas haloplanktis* | 5E-06 | 10 | bacteria |
| Ps281 | 1044 | hypothetical protein DEHA0F13321g | *Debaryomyces hansenii* | 6E-46 | 2 | yeast |
| Ps309 | 730 | Pre-mRNA splicing factor CWC21 | *Gibberella zeae* | 4E-17 | 2 | filamentous fungus |
| Ps_1284 | 680 | expressed protein | *Cryptococcus neoformans* | 2E-06 | 1 | yeast |
| Ps_213 | 687 | Pre-mRNA splicing factor CWC25 | *Gibberella zeae* | 2E-38 | 1 | filamentous fungus |
| Ps_2193 | 321 | intronic ORF at intron 1 of cox1 | *Crinipellis perniciosa* | 1E-06 | 1 | filamentous fungus |
| Ps_225 | 642 | small nuclear ribonucleoprotein hPrp3 | *Cryptococcus neoformans* | 3E-27 | 1 | yeast |
| Ps_3434 | 503 | similar to U6 snRNA-associated Sm-like protein LSm6 | *Schizosaccharomyces pombe* | 1e-22 | 1 | yeast |
| Ps_637 | 623 | hypothetical protein Lgas02000349 | *Lactobacillus gasseri* | 4E-09 | 1 | bacteria |
|  |  |  |  |  |  |  |
| 8 Transposon | |  |  |  |  |  |
| Ps49 | 684 | putative retroelement pol polyprotein | *Arabidopsis thaliana* | 2E-10 | 2 | plant |
|  |  |  |  |  |  |  |
| 9 Unclassified | |  |  |  |  |  |
| Ps1 | 673 | No hits found |  |  | 10 |  |
| Ps2 | 374 | No hits found |  |  | 2 |  |
| Ps3 | 297 | No hits found |  |  | 2 |  |
| Ps4 | 635 | No hits found |  |  | 2 |  |
| Ps5 | 397 | No hits found |  |  | 2 |  |
| Ps6 | 1469 | homologs of gas1 | *Magnaportha grisea* | 2E-06 | 4 | filamentous fungus |
| Ps7 | 547 | No hits found |  |  | 4 |  |
| Ps11 | 458 | unnamed protein product | *Candida glabrata* | 1E-10 | 3 | yeast |
| Additional file 4 (*continued*) | | | | | | |
| Uniseqs | Length(bp) | Description | Species | E value | No.of clones | Organism |
| Ps13 | 559 | No hits found |  |  | 2 |  |
| Ps14 | 316 | No hits found |  |  | 2 |  |
| Ps15 | 513 | No hits found |  |  | 2 |  |
| Ps16 | 640 | No hits found |  |  | 4 |  |
| Ps17 | 610 | No hits found |  |  | 2 |  |
| Ps18 | 290 | No hits found |  |  | 2 |  |
| Ps19 | 961 | No hits found |  |  | 9 |  |
| Ps20 | 598 | No hits found |  |  | 10 |  |
| Ps22 | 462 | hypothetical protein UM06051.1 | *Ustilago maydis* | 8E-18 | 2 | filamentous fungus |
| Ps23 | 756 | No hits found |  |  | 6 |  |
| Ps24 | 638 | No hits found |  |  | 182 |  |
| Ps26 | 721 | No hits found |  |  | 57 |  |
| Ps27 | 667 | hypothetical protein CND01180 | *Cryptococcus neoformans* | 3E-09 | 2 | yeast |
| Ps30 | 481 | hypothetical protein UM03836.1 | *Ustilago maydis* | 3E-14 | 2 | filamentous fungus |
| Ps31 | 1096 | No hits found |  |  | 7 |  |
| Ps32 | 561 | No hits found |  |  | 5 |  |
| Ps33 | 592 | No hits found |  |  | 4 |  |
| Ps35 | 592 | No hits found |  |  | 2 |  |
| Ps36 | 685 | No hits found |  |  | 4 |  |
| Ps37 | 683 | No hits found |  |  | 2 |  |
| Ps40 | 658 | hypothetical protein UM02926.1 | *Ustilago maydis* | 2E-17 | 2 | filamentous fungus |
| Ps42 | 504 | basal body protein NBP-2 | *Naegleria gruberi* | 7E-06 | 10 | animal |
| Ps43 | 631 | hypothetical protein UM05628.1 | *Ustilago maydis* | 4E-21 | 2 | filamentous fungus |
| Additional file 4 (*continued*) | | | | | | |
| Uniseqs | Length(bp) | Description | Species | E value | No.of clones | Organism |
| Ps44 | 673 | hypothetical protein FG08873.1 | *Gibberella zeae* | 8E-24 | 2 | filamentous fungus |
| Ps46 | 504 | No hits found |  |  | 2 |  |
| Ps47 | 456 | hypothetical protein DEHA0A10978g | *Debaryomyces hansenii* | 1E-17 | 2 | yeast |
| Ps48 | 716 | No hits found |  |  | 2 |  |
| Ps50 | 572 | No hits found |  |  | 10 |  |
| Ps51 | 477 | No hits found |  |  | 2 |  |
| Ps52 | 613 | No hits found |  |  | 5 |  |
| Ps54 | 1137 | No hits found |  |  | 7 |  |
| Ps56 | 448 | No hits found |  |  | 2 |  |
| Ps58 | 379 | No hits found |  |  | 2 |  |
| Ps59 | 518 | No hits found |  |  | 2 |  |
| Ps60 | 403 | No hits found |  |  | 4 |  |
| Ps61 | 1080 | No hits found |  |  | 2 |  |
| Ps63 | 310 | No hits found |  |  | 2 |  |
| Ps65 | 272 | No hits found |  |  | 2 |  |
| Ps66 | 711 | No hits found |  |  | 101 |  |
| Ps69 | 617 | No hits found |  |  | 2 |  |
| Ps70 | 389 | No hits found |  |  | 2 |  |
| Ps72 | 1036 | No hits found |  |  | 21 |  |
| Ps73 | 497 | No hits found |  |  | 2 |  |
| Ps76 | 705 | hypothetical protein CNL06480 | *Cryptococcus neoformans* | 3E-53 | 3 | yeast |
| Ps77 | 675 | No hits found |  |  | 61 |  |
| Ps78 | 2046 | unnamed protein product | *Kluyveromyces lactis* | 7E-27 | 29 | yeast |
| Additional file 4 (*continued*) | | | | | | |
| Uniseqs | Length(bp) | Description | Species | E value | No.of clones | Organism |
| Ps79 | 1072 | hypothetical protein | *Neurospora crassa* | 1E-10 | 11 | filamentous fungus |
| Ps80 | 674 | No hits found |  |  | 2 |  |
| Ps81 | 578 | hypothetical protein UM00581.1 | *Ustilago maydis* | 5E-16 | 3 | filamentous fungus |
| Ps82 | 405 | No hits found |  |  | 2 |  |
| Ps84 | 701 | No hits found |  |  | 11 |  |
| Ps87 | 456 | hesp-767 | *Melampsora lini* | 9E-30 | 2 | filamentous fungus |
| Ps90 | 608 | No hits found |  |  | 2 |  |
| Ps91 | 664 | No hits found |  |  | 4 |  |
| Ps95 | 714 | No hits found |  |  | 11 |  |
| Ps97 | 630 | No hits found |  |  | 2 |  |
| Ps98 | 691 | No hits found |  |  | 8 |  |
| Ps99 | 835 | No hits found |  |  | 7 |  |
| Ps102 | 535 | No hits found |  |  | 2 |  |
| Ps103 | 658 | No hits found |  |  | 7 |  |
| Ps104 | 851 | hypothetical protein UM04715.1 | *Ustilago maydis* | 1E-30 | 5 | filamentous fungus |
| Ps106 | 374 | No hits found |  |  | 2 |  |
| Ps107 | 630 | No hits found |  |  | 6 |  |
| Ps108 | 829 | No hits found |  |  | 39 |  |
| Ps109 | 677 | No hits found |  |  | 2 |  |
| Ps110 | 705 | No hits found |  |  | 2 |  |
| Ps111 | 484 | No hits found |  |  | 13 |  |
| Ps114 | 655 | No hits found |  |  | 3 |  |
| Ps115 | 1288 | No hits found |  |  | 20 |  |
| Additional file 4 (*continued*) | | | | | | |
| Uniseqs | Length(bp) | Description | Species | E value | No.of clones | Organism |
| Ps117 | 528 | hypothetical protein UM05487.1 | *Ustilago maydis* | 7E-09 | 3 | filamentous fungus |
| Ps118 | 883 | No hits found |  |  | 6 |  |
| Ps120 | 739 | No hits found |  |  | 5 |  |
| Ps123 | 922 | No hits found |  |  | 23 |  |
| Ps125 | 497 | No hits found |  |  | 4 |  |
| Ps126 | 637 | No hits found |  |  | 21 |  |
| Ps127 | 561 | No hits found |  |  | 2 |  |
| Ps129 | 693 | No hits found |  |  | 121 |  |
| Ps130 | 482 | No hits found |  |  | 3 |  |
| Ps132 | 656 | No hits found |  |  | 28 |  |
| Ps133 | 873 | No hits found |  |  | 10 |  |
| Ps134 | 650 | No hits found |  |  | 20 |  |
| Ps135 | 546 | No hits found |  |  | 2 |  |
| Ps136 | 631 | No hits found |  |  | 2 |  |
| Ps137 | 391 | No hits found |  |  | 15 |  |
| Ps138 | 1141 | No hits found |  |  | 19 |  |
| Ps141 | 740 | No hits found |  |  | 16 |  |
| Ps142 | 351 | No hits found |  |  | 2 |  |
| Ps143 | 625 | No hits found |  |  | 3 |  |
| Ps144 | 656 | No hits found |  |  | 3 |  |
| Ps146 | 421 | No hits found |  |  | 2 |  |
| Ps147 | 684 | No hits found |  |  | 2 |  |
| Ps148 | 542 | No hits found |  |  | 2 |  |
| Additional file 4 (*continued*) | | | | | | |
| Uniseqs | Length(bp) | Description | Species | E value | No.of clones | Organism |
| Ps150 | 1499 | No hits found |  |  | 6 |  |
| Ps152 | 702 | No hits found |  |  | 50 |  |
| Ps155 | 1024 | No hits found |  |  | 19 |  |
| Ps156 | 503 | No hits found |  |  | 2 |  |
| Ps157 | 1277 | No hits found |  |  | 6 |  |
| Ps158 | 758 | No hits found |  |  | 5 |  |
| Ps159 | 1108 | homologs of gas2 | *Magnaportha grisea* | 1E-08 | 19 | filamentous fungus |
| Ps160 | 1113 | hypothetical protein UM01629.1 | *Ustilago maydis* | 2E-14 | 57 | filamentous fungus |
| Ps161 | 644 | hypothetical protein UM03538. | *Ustilago maydis* | 3E-38 | 2 | filamentous fungus |
| Ps162 | 674 | No hits found |  |  | 5 |  |
| Ps163 | 865 | protein targeting-related protein, putative | *Cryptococcusneoformans* | 6E-30 | 2 | yeast |
| Ps165 | 501 | No hits found |  |  | 3 |  |
| Ps166 | 969 | No hits found |  |  | 5 |  |
| Ps167 | 693 | No hits found |  |  | 6 |  |
| Ps170 | 864 | hypothetical protein Afu3g08990 | *Aspergillus fumigatus* | 2E-11 | 8 | filamentous fungus |
| Ps171 | 620 | No hits found |  |  | 56 |  |
| Ps173 | 476 | No hits found |  |  | 2 |  |
| Ps174 | 707 | No hits found |  |  | 2 |  |
| Ps175 | 401 | No hits found |  |  | 2 |  |
| Ps178 | 357 | No hits found |  |  | 2 |  |
| Ps179 | 359 | No hits found |  |  | 2 |  |
| Ps180 | 299 | No hits found |  |  | 4 |  |
| Ps181 | 793 | No hits found |  |  | 4 |  |
| Additional file 4 (*continued*) | | | | | | |
| Uniseqs | Length(bp) | Description | Species | E value | No.of clones | Organism |
| Ps182 | 329 | No hits found |  |  | 2 |  |
| Ps187 | 334 | No hits found |  |  | 3 |  |
| Ps188 | 477 | No hits found |  |  | 2 |  |
| Ps191 | 633 | No hits found |  |  | 2 |  |
| Ps192 | 573 | No hits found |  |  | 3 |  |
| Ps193 | 714 | No hits found |  |  | 4 |  |
| Ps194 | 514 | No hits found |  |  | 3 |  |
| Ps195 | 582 | No hits found |  |  | 20 |  |
| Ps196 | 433 | No hits found |  |  | 11 |  |
| Ps197 | 688 | No hits found |  |  | 4 |  |
| Ps198 | 654 | No hits found |  |  | 122 |  |
| Ps199 | 690 | No hits found |  |  | 2 |  |
| Ps202 | 704 | No hits found |  |  | 4 |  |
| Ps203 | 634 | No hits found |  |  | 2 |  |
| Ps204 | 886 | No hits found |  |  | 25 |  |
| Ps207 | 526 | hypothetical protein | *Yarrowia lipolytica* | 9E-07 | 5 | plant |
| Ps208 | 742 | No hits found |  |  | 2 |  |
| Ps210 | 564 | expressed protein | *Cryptococcus neoformans* | 2E-16 | 3 | yeast |
| Ps211 | 626 | hypothetical protein UM01811.1 | *Ustilago maydis* | 1E-28 | 3 | filamentous fungus |
| Ps212 | 1068 | No hits found |  |  | 9 |  |
| Ps213 | 568 | No hits found |  |  | 2 |  |
| Ps214 | 632 | No hits found |  |  | 2 |  |
| Ps216 | 516 | No hits found |  |  | 2 |  |
| Additional file 4 (*continued*) | | | | | | |
| Uniseqs | Length(bp) | Description | Species | E value | No.of clones | Organism |
| Ps217 | 610 | No hits found |  |  | 2 |  |
| Ps218 | 369 | hypothetical protein | *Macaca fascicularis* | 1E-06 | 3 | animal |
| Ps219 | 652 | No hits found |  |  | 63 |  |
| Ps220 | 739 | No hits found |  |  | 2 |  |
| Ps221 | 910 | hypothetical protein AN3310.2 | *Aspergillus nidulans* | 3E-15 | 2 | filamentous fungus |
| Ps222 | 389 | No hits found |  |  | 2 |  |
| Ps223 | 604 | No hits found |  |  | 3 |  |
| Ps224 | 1153 | No hits found |  |  | 7 |  |
| Ps225 | 536 | hypothetical protein CNL05720 | *Cryptococcus neoformans* | 1E-09 | 2 | yeast |
| Ps226 | 383 | No hits found |  |  | 4 |  |
| Ps230 | >719 | hypothetical protein DDB0206177 | *Dictyostelium discoideum* | 2E-08 | 5 | filamentous fungus |
| Ps231 | 1030 | No hits found |  |  | 38 |  |
| Ps233 | 635 | No hits found |  |  | 2 |  |
| Ps234 | 650 | hypothetical protein UM06133.1 | *Ustilago maydis* | 4E-29 | 2 | filamentous fungus |
| Ps235 | 839 | hypothetical protein UM04352.1 | *Ustilago maydis* | 5E-86 | 4 | filamentous fungus |
| Ps236 | 645 | No hits found |  |  | 2 |  |
| Ps237 | 571 | priA | *Lentinula edodes* | 7E-14 | 5 | filamentous fungus |
| Ps239 | 1024 | No hits found |  |  | 36 |  |
| Ps240 | 463 | No hits found |  |  | 2 |  |
| Ps241 | 1037 | No hits found |  |  | 13 |  |
| Ps242 | 650 | XRnf12C | *Xenopus laevis* | 4E-10 | 5 | animal |
| Ps243 | 405 | No hits found |  |  | 2 |  |
| Ps244 | 496 | No hits found |  |  | 11 |  |
| Additional file 4 (*continued*) | | | | | | |
| Uniseqs | Length(bp) | Description | Species | E value | No.of clones | Organism |
| Ps245 | 1053 | No hits found |  |  | 5 |  |
| Ps246 | 416 | hypothetical protein AN2876.2 | *Aspergillus nidulans* | 1E-37 | 2 | filamentous fungus |
| Ps247 | 647 | similar to ENSANGP00000015673 | *Apis mellifera* | 4E-33 | 2 | animal |
| Ps251 | 549 | No hits found |  |  | 7 |  |
| Ps252 | 657 | No hits found |  |  | 127 |  |
| Ps253 | 703 | putative secreted protein | *Ixodes scapularis* | 9E-06 | 110 | insect |
| Ps254 | 724 | No hits found |  |  | 3 |  |
| Ps255 | 610 | No hits found |  |  | 2 |  |
| Ps256 | 600 | No hits found |  |  | 3 |  |
| Ps257 | 570 | hypothetical protein | *Neurospora crassa* | 8E-18 | 2 | filamentous fungus |
| Ps258 | 732 | No hits found |  |  | 7 |  |
| Ps265 | 305 | No hits found |  |  | 2 |  |
| Ps267 | 587 | putative secreted protein | *Ixodes scapularis* | 2E-06 | 3 | insect |
| Ps269 | 587 | No hits found |  |  | 2 |  |
| Ps274 | 655 | unnamed protein product | *Aspergillus oryzae* | 2E-16 | 3 | filamentous fungus |
| Ps275 | 451 | No hits found |  |  | 2 |  |
| Ps277 | 421 | No hits found |  |  | 2 |  |
| Ps279 | 648 | hypothetical protein AN8695.2 | *Aspergillus nidulans* | 2E-15 | 2 | filamentous fungus |
| Ps280 | 674 | hypothetical protein UM01311.1 | *Ustilago maydis* | 8E-38 | 2 | filamentous fungus |
| Ps284 | 295 | No hits found |  |  | 3 |  |
| Ps285 | 608 | hypothetical protein UM02937.1 | *Ustilago maydis* | 2E-12 | 2 | filamentous fungus |
| Ps286 | 919 | allergen | *Malassezia sympodialis* | 2E-28 | 6 | filamentous fungus |
| Ps292 | 596 | No hits found |  |  | 7 |  |
| Additional file 4 (*continued*) | | | | | | |
| Uniseqs | Length(bp) | Description | Species | E value | No.of clones | Organism |
| Ps294 | 234 | No hits found |  |  | 7 |  |
| Ps295 | 678 | No hits found |  |  | 4 |  |
| Ps298 | 680 | hypothetical protein UM06082.1 | *Ustilago maydis* | 2E-61 | 2 | filamentous fungus |
| Ps304 | 754 | No hits found |  |  | 2 |  |
| Ps307 | 660 | No hits found |  |  | 3 |  |
| Ps308 | 610 | No hits found |  |  | 2 |  |
| Ps310 | 636 | priA | *Lentinula edodes* | 1E-12 | 2 | filamentous fungus |
| Ps311 | 1524 | Hypothetical protein Franean1DRAFT_6526 | *Frankia sp. EAN1pec* | 1E-11 | 35 | 放线菌 |
| Ps313 | 489 | No hits found |  |  | 2 |  |
| Ps_1 | 683 | hypothetical protein | *Neurospora crassa* | 5E-43 | 1 | filamentous fungus |
| Ps_3 | 180 | PREDICTED: hypothetical protein XP_580193 | *Rattus norvegicus* | 3E-06 | 1 | animal |
| Ps_4 | 582 | No hits found |  |  | 1 |  |
| Ps_19 | 232 | No hits found |  |  | 1 |  |
| Ps_34 | 392 | No hits found |  |  | 1 |  |
| Ps_38 | 770 | No hits found |  |  | 1 |  |
| Ps_49 | 656 | hypothetical protein UM00961.1 | *Ustilago maydis* | 2E-44 | 1 | filamentous fungus |
| Ps_84 | 669 | hypothetical protein | *Yarrowia lipolytica* | 4E-55 | 1 | plant |
| Ps_96 | 631 | No hits found |  |  | 1 |  |
| Ps_111 | 472 | No hits found |  |  | 1 |  |
| Ps_122 | 321 | No hits found |  |  | 1 |  |
| Ps_123 | 708 | No hits found |  |  | 1 |  |
| Ps_124 | 425 | No hits found |  |  | 1 |  |
| Ps_125 | 439 | hypothetical protein XP_580193 | *Rattus norvegicus* | 1E-10 | 1 | animal |
| Additional file 4 (*continued*) | | | | | | |
| Uniseqs | Length(bp) | Description | Species | E value | No.of clones | Organism |
| Ps_127 | 740 | No hits found |  |  | 1 |  |
| Ps_129 | 279 | No hits found |  |  | 1 |  |
| Ps_139 | 764 | hypothetical protein UM02957.1 | *Ustilago maydis* | 2E-58 | 1 | filamentous fungus |
| Ps_141 | 405 | hypothetical protein | *Oryza sativa* | 2E-08 | 1 | plant |
| Ps_164 | 318 | No hits found |  |  | 1 |  |
| Ps_202 | 324 | No hits found |  |  | 1 |  |
| Ps_214 | 331 | No hits found |  |  | 1 |  |
| Ps_222 | 628 | No hits found |  |  | 1 |  |
| Ps_240 | 782 | hypothetical protein | *Yarrowia lipolytica* | 2E-24 | 1 | plant |
| Ps_243 | 501 | No hits found |  |  | 1 |  |
| Ps_247 | 635 | No hits found |  |  | 1 |  |
| Ps_256 | 382 | No hits found |  |  | 1 |  |
| Ps_260 | 713 | hypothetical protein UM04672.1 | *Ustilago maydis* | 6E-10 | 1 | filamentous fungus |
| Ps_267 | 443 | No hits found |  |  | 1 |  |
| Ps_302 | 322 | No hits found |  |  | 1 |  |
| Ps_314 | 505 | No hits found |  |  | 1 |  |
| Ps_317 | 450 | No hits found |  |  | 1 |  |
| Ps_333 | 519 | unnamed protein product | *Kluyveromyces lactis* | 4E-11 | 1 | yeast |
| Ps_335 | 577 | No hits found |  |  | 1 |  |
| Ps_336 | 314 | No hits found |  |  | 1 |  |
| Ps_354 | 410 | No hits found |  |  | 1 |  |
| Ps_357 | 482 | No hits found |  |  | 1 |  |
| Ps_368 | 254 | No hits found |  |  | 1 |  |
| Additional file 4 (*continued*) | | | | | | |
| Uniseqs | Length(bp) | Description | Species | E value | No.of clones | Organism |
| Ps_371 | 306 | No hits found |  |  | 1 |  |
| Ps_373 | 499 | No hits found |  |  | 1 |  |
| Ps_385 | 507 | No hits found |  |  | 1 |  |
| Ps_391 | 413 | hypothetical protein | *Oryza sativa* | 2E-08 | 1 | plant |
| Ps_401 | 408 | No hits found |  |  | 1 |  |
| Ps_422 | 409 | hypothetical protein | *Oryza sativa* | 2E-08 | 1 | plant |
| Ps_428 | 496 | No hits found |  |  | 1 |  |
| Ps_442 | 459 | No hits found |  |  | 1 |  |
| Ps_449 | 755 | No hits found |  |  | 1 |  |
| Ps_452 | 655 | No hits found |  |  | 1 |  |
| Ps_455 | 320 | No hits found |  |  | 1 |  |
| Ps_466 | 712 | expressed protein | *Cryptococcus neoformans* | 1E-38 | 1 | yeast |
| Ps_473 | 515 | No hits found |  |  | 1 |  |
| Ps_476 | 317 | No hits found |  |  | 1 |  |
| Ps_484 | 527 | No hits found |  |  | 1 |  |
| Ps_486 | 684 | No hits found |  |  | 1 |  |
| Ps_488 | 470 | similar to hypothetical protein | *Rattus norvegicus* | 1E-23 | 1 | animal |
| Ps_499 | 402 | No hits found |  |  | 1 |  |
| Ps_502 | 401 | No hits found |  |  | 1 |  |
| Ps_507 | 703 | No hits found |  |  | 1 |  |
| Ps_510 | 771 | No hits found |  |  | 1 |  |
| Ps_512 | 704 | No hits found |  |  | 1 |  |
| Ps_515 | 485 | No hits found |  |  | 1 |  |
| Additional file 4 (*continued*) | | | | | | |
| Uniseqs | Length(bp) | Description | Species | E value | No.of clones | Organism |
| Ps_522 | 773 | No hits found |  |  | 1 |  |
| Ps_523 | 381 | hypothetical protein | *Oryza sativa* | 2E-07 | 1 | plant |
| Ps_551 | 403 | hypothetical protein | *Oryza sativa* | 2E-08 | 1 | plant |
| Ps_562 | 455 | No hits found |  |  | 1 |  |
| Ps_580 | 375 | No hits found |  |  | 1 |  |
| Ps_583 | 447 | hypothetical protein XP_580193 | *Rattus norvegicus* | 1E-10 | 1 | animal |
| Ps_592 | 446 | Hypothetical protein W09C5.1 | *Caenorhabditis elegans* | 1E-17 | 1 | animal |
| Ps_613 | 577 | No hits found |  |  | 1 |  |
| Ps_614 | 397 | No hits found |  |  | 1 |  |
| Ps_616 | 722 | No hits found |  |  | 1 |  |
| Ps_617 | 589 | No hits found |  |  | 1 |  |
| Ps_626 | 309 | No hits found |  |  | 1 |  |
| Ps_627 | 300 | No hits found |  |  | 1 |  |
| Ps_636 | 321 | No hits found |  |  | 1 |  |
| Ps_643 | 470 | No hits found |  |  | 1 |  |
| Ps_665 | 436 | No hits found |  |  | 1 |  |
| Ps_667 | 382 | No hits found |  |  | 1 |  |
| Ps_668 | 181 | No hits found |  |  | 1 |  |
| Ps_670 | 426 | hypothetical protein | *Oryza sativa* | 2E-08 | 1 | plant |
| Ps_672 | 197 | hypothetical protein XP_580193 | *Rattus norvegicus* | 2E-10 | 1 | animal |
| Ps_673 | 182 | No hits found |  |  | 1 |  |
| Ps_674 | 498 | basal body protein NBP-2 | *Naegleria gruberi* | 7E-06 | 1 | animal |
| Ps_677 | 785 | hypothetical protein AN2344.2 | *Aspergillus nidulans* | 2E-45 | 1 | filamentous fungus |
| Additional file 4 (*continued*) | | | | | | |
| Uniseqs | Length(bp) | Description | Species | E value | No.of clones | Organism |
| Ps_678 | 429 | No hits found |  |  | 1 |  |
| Ps_680 | 294 | No hits found |  |  | 1 |  |
| Ps_682 | 440 | No hits found |  |  | 1 |  |
| Ps_693 | 578 | No hits found |  |  | 1 |  |
| Ps_707 | 283 | No hits found |  |  | 1 |  |
| Ps_716 | 320 | No hits found |  |  | 1 |  |
| Ps_718 | 344 | No hits found |  |  | 1 |  |
| Ps_724 | 658 | hypothetical protein Afu6g04430 | *Aspergillus fumigatus* | 2E-52 | 1 | filamentous fungus |
| Ps_730 | 206 | No hits found |  |  | 1 |  |
| Ps_734 | 407 | hypothetical protein | *Oryza sativa* | 7E-09 | 1 | plant |
| Ps_735 | 603 | No hits found |  |  | 1 |  |
| Ps_739 | 543 | No hits found |  |  | 1 |  |
| Ps_742 | 232 | No hits found |  |  | 1 |  |
| Ps_758 | 242 | No hits found |  |  | 1 |  |
| Ps_763 | 185 | hypothetical protein XP_580193 | *Rattus norvegicus* | 2E-07 | 1 | animal |
| Ps_778 | 252 | No hits found |  |  | 1 |  |
| Ps_779 | 271 | conserved hypothetical protein | *Chaetomium globosum* | 1E-18 | 1 | filamentous fungus |
| Ps_783 | 196 | hypothetical protein XP_580193 | *Rattus norvegicus* | 2E-10 | 1 | animal |
| Ps_786 | 263 | No hits found |  |  | 1 |  |
| Ps_789 | 361 | No hits found |  |  | 1 |  |
| Ps_800 | 656 | No hits found |  |  | 1 |  |
| Ps_801 | 626 | No hits found |  |  | 1 |  |
| Ps_803 | 206 | No hits found |  |  | 1 |  |
| Additional file 4 (*continued*) | | | | | | |
| Uniseqs | Length(bp) | Description | Species | E value | No.of clones | Organism |
| Ps_811 | 176 | unnamed protein product | *Kluyveromyces lactis* | 4E-08 | 1 | yeast |
| Ps_815 | 456 | No hits found |  |  | 1 |  |
| Ps_818 | 653 | No hits found |  |  | 1 |  |
| Ps_819 | 541 | No hits found |  |  | 1 |  |
| Ps_842 | 521 | hypothetical protein UM03218.1 | *Ustilago maydis* | 8E-08 | 1 | filamentous fungus |
| Ps_855 | 664 | hypothetical protein UM03349.1 | *Ustilago maydis* | 4E-42 | 1 | filamentous fungus |
| Ps_861 | 328 | No hits found |  |  | 1 |  |
| Ps_874 | 576 | No hits found |  |  | 1 |  |
| Ps_879 | 150 | unnamed protein product | *Kluyveromyces lactis* | 6E-08 | 1 | yeast |
| Ps_880 | 204 | No hits found |  |  | 1 |  |
| Ps_886 | 322 | No hits found |  |  | 1 |  |
| Ps_889 | 244 | No hits found |  |  | 1 |  |
| Ps_893 | 310 | No hits found |  |  | 1 |  |
| Ps_916 | 310 | No hits found |  |  | 1 |  |
| Ps_927 | 272 | No hits found |  |  | 1 |  |
| Ps_935 | 429 | unnamed protein product | *Kluyveromyces lactis* | 3E-08 | 1 | yeast |
| Ps_936 | 254 | No hits found |  |  | 1 |  |
| Ps_940 | 719 | No hits found |  |  | 1 |  |
| Ps_941 | 595 | hypothetical protein | *Yarrowia lipolytica* | 1E-10 | 1 | plant |
| Ps_951 | 318 | No hits found |  |  | 1 |  |
| Ps_957 | 185 | unknown | *Saccharomyces cerevisiae* | 5E-07 | 1 | yeast |
| Ps_959 | 169 | hypothetical protein XP_580193 | *Rattus norvegicus* | 2E-07 | 1 | animal |
| Ps_960 | 170 | hypothetical protein XP_580193 | *Rattus norvegicus* | 1E-10 | 1 | animal |
| Additional file 4 (*continued*) | | | | | | |
| Uniseqs | Length(bp) | Description | Species | E value | No.of clones | Organism |
| Ps_965 | 413 | No hits found |  |  | 1 |  |
| Ps_994 | 383 | No hits found |  |  | 1 |  |
| Ps_1001 | 725 | hypothetical protein UM02880.1 | *Ustilago maydis* | 2E-06 | 1 | filamentous fungus |
| Ps_1016 | 329 | No hits found |  |  | 1 |  |
| Ps_1021 | 498 | No hits found |  |  | 1 |  |
| Ps_1029 | 251 | No hits found |  |  | 1 |  |
| Ps_1037 | 371 | No hits found |  |  | 1 |  |
| Ps_1039 | 314 | No hits found |  |  | 1 |  |
| Ps_1046 | 378 | No hits found |  |  | 1 |  |
| Ps_1050 | 348 | No hits found |  |  | 1 |  |
| Ps_1053 | 427 | No hits found |  |  | 1 |  |
| Ps_1056 | 220 | No hits found |  |  | 1 |  |
| Ps_1059 | 269 | No hits found |  |  | 1 |  |
| Ps_1067 | 314 | No hits found |  |  | 1 |  |
| Ps_1069 | 373 | No hits found |  |  | 1 |  |
| Ps_1070 | 203 | No hits found |  |  | 1 |  |
| Ps_1071 | 759 | hypothetical protein UM06073.1 | *Ustilago maydis* | 9E-21 | 1 | filamentous fungus |
| Ps_1076 | 323 | No hits found |  |  | 1 |  |
| Ps_1101 | 323 | No hits found |  |  | 1 |  |
| Ps_1126 | 322 | No hits found |  |  | 1 |  |
| Ps_1127 | 191 | No hits found |  |  | 1 |  |
| Ps_1135 | 259 | No hits found |  |  | 1 |  |
| Ps_1141 | 238 | No hits found |  |  | 1 |  |
| Additional file 4 (*continued*) | | | | | | |
| Uniseqs | Length(bp) | Description | Species | E value | No.of clones | Organism |
| Ps_1142 | 696 | hypothetical protein UM01108.1 | *Ustilago maydis* | 2E-43 | 1 | filamentous fungus |
| Ps_1151 | 751 | No hits found |  |  | 1 |  |
| Ps_1164 | 738 | No hits found |  |  | 1 |  |
| Ps_1167 | 316 | No hits found |  |  | 1 |  |
| Ps_1182 | 336 | No hits found |  |  | 1 |  |
| Ps_1191 | 726 | No hits found |  |  | 1 |  |
| Ps_1198 | 475 | No hits found |  |  | 1 |  |
| Ps_1203 | 372 | No hits found |  |  | 1 |  |
| Ps_1204 | 603 | No hits found |  |  | 1 |  |
| Ps_1206 | 314 | conserved hypothetical protein | *Chaetomium globosum* | 4E-18 | 1 | filamentous fungus |
| Ps_1222 | 519 | hypothetical protein UM06336.1 | *Ustilago maydis* | 1E-23 | 1 | filamentous fungus |
| Ps_1265 | 314 | No hits found |  |  | 1 |  |
| Ps_1279 | 434 | No hits found |  |  | 1 |  |
| Ps_1290 | 508 | No hits found |  |  | 1 |  |
| Ps_1315 | 216 | No hits found |  |  | 1 |  |
| Ps_1355 | 365 | No hits found |  |  | 1 |  |
| Ps_1370 | 227 | No hits found |  |  | 1 |  |
| Ps_1371 | 524 | No hits found |  |  | 1 |  |
| Ps_1373 | 639 | hypothetical protein UM03931.1 | *Ustilago maydis* | 1E-06 | 1 | filamentous fungus |
| Ps_1398 | 228 | No hits found |  |  | 1 |  |
| Ps_1441 | 310 | No hits found |  |  | 1 |  |
| Ps_1442 | 313 | No hits found |  |  | 1 |  |
| Ps_1465 | 299 | No hits found |  |  | 1 |  |
| Additional file 4 (*continued*) | | | | | | |
| Uniseqs | Length(bp) | Description | Species | E value | No.of clones | Organism |
| Ps_1466 | 291 | No hits found |  |  | 1 |  |
| Ps_1480 | 112 | No hits found |  |  | 1 |  |
| Ps_1482 | 320 | No hits found |  |  | 1 |  |
| Ps_1484 | 614 | hypothetical protein UM01767.1 | *Ustilago maydis* | 2E-07 | 1 | filamentous fungus |
| Ps_1487 | 265 | No hits found |  |  | 1 |  |
| Ps_1497 | 411 | No hits found |  |  | 1 |  |
| Ps_1499 | 122 | No hits found |  |  | 1 |  |
| Ps_1512 | 265 | No hits found |  |  | 1 |  |
| Ps_1513 | 221 | No hits found |  |  | 1 |  |
| Ps_1517 | 592 | hypothetical protein UM02800.1 | *Ustilago maydis* | 3E-12 | 1 | filamentous fungus |
| Ps_1545 | 323 | No hits found |  |  | 1 |  |
| Ps_1552 | 128 | No hits found |  |  | 1 |  |
| Ps_1563 | 153 | No hits found |  |  | 1 |  |
| Ps_1564 | 365 | No hits found |  |  | 1 |  |
| Ps_1571 | 575 | hypothetical protein DEHA0C01441g | *Debaryomyces hansenii* | 1E-14 | 1 | yeast |
| Ps_1582 | 342 | No hits found |  |  | 1 |  |
| Ps_1597 | 642 | conserved hypothetical protein | *Cryptococcus neoformans* | 2E-11 | 1 | yeast |
| Ps_1622 | 679 | No hits found |  |  | 1 |  |
| Ps_1649 | 469 | expressed protein | *Cryptococcus neoformans* | 1E-07 | 1 | yeast |
| Ps_1657 | 409 | No hits found |  |  | 1 |  |
| Ps_1672 | 362 | No hits found |  |  | 1 |  |
| Ps_1673 | 353 | No hits found |  |  | 1 |  |
| Ps_1739 | 554 | No hits found |  |  | 1 |  |
| Additional file 4 (*continued*) | | | | | | |
| Uniseqs | Length(bp) | Description | Species | E value | No.of clones | Organism |
| Ps_1746 | 499 | No hits found |  |  | 1 |  |
| Ps_1774 | 505 | hypothetical protein DEHA0C01441g | *Debaryomyces hansenii* | 1E-14 | 1 | yeast |
| Ps_1775 | 307 | No hits found |  |  | 1 |  |
| Ps_1787 | 353 | No hits found |  |  | 1 |  |
| Ps_1794 | 273 | No hits found |  |  | 1 |  |
| Ps_1797 | 353 | hypothetical protein UM02579.1 | *Ustilago maydis* | 2E-13 | 1 | filamentous fungus |
| Ps_1804 | 481 | No hits found |  |  | 1 |  |
| Ps_1821 | 342 | No hits found |  |  | 1 |  |
| Ps_1835 | 335 | No hits found |  |  | 1 |  |
| Ps_1843 | 600 | No hits found |  |  | 1 |  |
| Ps_184 | 323 | No hits found |  |  | 1 |  |
| Ps_1872 | 244 | No hits found |  |  | 1 |  |
| Ps_1874 | 403 | hypothetical protein | *Oryza sativa* | 2E-08 | 1 | plant |
| Ps_1892 | 432 | No hits found |  |  | 1 |  |
| Ps_1906 | 419 | No hits found |  |  | 1 |  |
| Ps_1913 | 467 | No hits found |  |  | 1 |  |
| Ps_1930 | 427 | No hits found |  |  | 1 |  |
| Ps_1932 | 452 | hypothetical protein UM01150.1 | *Ustilago maydis* | 1E-07 | 1 | filamentous fungus |
| Ps_1935 | 420 | No hits found |  |  | 1 |  |
| Ps_1943 | 343 | hypothetical protein XP_580193 | *Rattus norvegicus* | 1E-10 | 1 | animal |
| Ps_1944 | 316 | No hits found |  |  | 1 |  |
| Ps_1948 | 640 | No hits found |  |  | 1 |  |
| Ps_1949 | 366 | No hits found |  |  | 1 |  |
| Additional file 4 (*continued*) | | | | | | |
| Uniseqs | Length(bp) | Description | Species | E value | No.of clones | Organism |
| Ps_1956 | 403 | hypothetical protein | *Oryza sativa* | 2E-08 | 1 | plant |
| Ps_1977 | 399 | hypothetical protein | *Oryza sativa* | 2E-08 | 1 | plant |
| Ps_1986 | 317 | No hits found |  |  | 1 |  |
| Ps_1996 | 371 | hypothetical protein UM04550.1 | *Ustilago maydis* | 1E-11 | 1 | filamentous fungus |
| Ps_2003 | 171 | hypothetical protein XP_580193 | *Rattus norvegicus* | 1E-07 | 1 | animal |
| Ps_2004 | 307 | No hits found |  |  | 1 |  |
| Ps_2011 | 415 | No hits found |  |  | 1 |  |
| Ps_2014 | 189 | No hits found |  |  | 1 |  |
| Ps_2042 | 344 | No hits found |  |  | 1 |  |
| Ps_2043 | 322 | No hits found |  |  | 1 |  |
| Ps_2044 | 633 | Bromodomain and PHD finger-containing protein 3 | *Cryptococcus neoformans* | 4E-20 | 1 | yeast |
| Ps_2045 | 247 | No hits found |  |  | 1 |  |
| Ps_2052 | 200 | No hits found |  |  | 1 |  |
| Ps_2078 | 179 | No hits found |  |  | 1 |  |
| Ps_2079 | 236 | No hits found |  |  | 1 |  |
| Ps_2083 | 735 | No hits found |  |  | 1 |  |
| Ps_2093 | 174 | hypothetical protein XP_580193 | *Rattus norvegicus* | 1E-10 | 1 | animal |
| Ps_2098 | 571 | No hits found |  |  | 1 |  |
| Ps_2100 | 262 | No hits found |  |  | 1 |  |
| Ps_2101 | 484 | No hits found |  |  | 1 |  |
| Ps_2105 | 171 | hypothetical protein XP_580193 | *Rattus norvegicus* | 5E-09 | 1 | animal |
| Ps_2106 | 608 | No hits found |  |  | 1 |  |
| Ps_2121 | 650 | No hits found |  |  | 1 |  |
| Additional file 4 (*continued*) | | | | | | |
| Uniseqs | Length(bp) | Description | Species | E value | No.of clones | Organism |
| Ps_2130 | 320 | No hits found |  |  | 1 |  |
| Ps_2139 | 228 | No hits found |  |  | 1 |  |
| Ps_2185 | 168 | No hits found |  |  | 1 |  |
| Ps_2194 | 309 | No hits found |  |  | 1 |  |
| Ps_2198 | 342 | No hits found |  |  | 1 |  |
| Ps_2202 | 401 | No hits found |  |  | 1 |  |
| Ps_2203 | 397 | No hits found |  |  | 1 |  |
| Ps_2238 | 362 | No hits found |  |  | 1 |  |
| Ps_2242 | 541 | hypothetical protein SPAC31G5.21 | *Schizosaccharomyces pombe* | 2E-11 | 1 | yeast |
| Ps_2267 | 491 | No hits found |  |  | 1 |  |
| Ps_2274 | 202 | No hits found |  |  | 1 |  |
| Ps_2285 | 347 | No hits found |  |  | 1 |  |
| Ps_2297 | 650 | hypothetical protein CNBE0540 | *Cryptococcus neoformans* | 3E-08 | 1 | yeast |
| Ps_2316 | 390 | No hits found |  |  | 1 |  |
| Ps_2324 | 289 | No hits found |  |  | 1 |  |
| Ps_2325 | 215 | No hits found |  |  | 1 |  |
| Ps_2329 | 500 | No hits found |  |  | 1 |  |
| Ps_2336 | 309 | No hits found |  |  | 1 |  |
| Ps_2341 | 370 | No hits found |  |  | 1 |  |
| Ps_2343 | 394 | No hits found |  |  | 1 |  |
| Ps_2350 | 341 | No hits found |  |  | 1 |  |
| Ps_2418 | 357 | No hits found |  |  | 1 |  |
| Ps_2419 | 455 | No hits found |  |  | 1 |  |
| Additional file 4 (*continued*) | | | | | | |
| Uniseqs | Length(bp) | Description | Species | E value | No.of clones | Organism |
| Ps_2437 | 435 | No hits found |  |  | 1 |  |
| Ps_2444 | 266 | No hits found |  |  | 1 |  |
| Ps_2459 | 569 | No hits found |  |  | 1 |  |
| Ps_2469 | 318 | No hits found |  |  | 1 |  |
| Ps_2480 | 585 | No hits found |  |  | 1 |  |
| Ps_2488 | 181 | hypothetical protein XP_580193 | *Rattus norvegicus* | 1E-08 | 1 | animal |
| Ps_2495 | 278 | No hits found |  |  | 1 |  |
| Ps_2502 | 497 | No hits found |  |  | 1 |  |
| Ps_2517 | 323 | No hits found |  |  | 1 |  |
| Ps_2689 | 336 | No hits found |  |  | 1 |  |
| Ps_2709 | 375 | No hits found |  |  | 1 |  |
| Ps_2717 | 557 | No hits found |  |  | 1 |  |
| Ps_2719 | 345 | No hits found |  |  | 1 |  |
| Ps_2733 | 389 | hypothetical protein | *Oryza sativa* | 2E-08 | 1 | plant |
| Ps_2746 | 577 | No hits found |  |  | 1 |  |
| Ps_2751 | 591 | No hits found |  |  | 1 |  |
| Ps_2760 | 530 | No hits found |  |  | 1 |  |
| Ps_2769 | 458 | unnamed protein product | *Aspergillus oryzae* | 3E-11 | 1 | filamentous fungus |
| Ps_2778 | 312 | No hits found |  |  | 1 |  |
| Ps_2786 | 505 | No hits found |  |  | 1 |  |
| Ps_2796 | 268 | hypothetical protein XP_580193 | *Rattus norvegicus* | 1E-07 | 1 | animal |
| Ps_2803 | 395 | No hits found |  |  | 1 |  |
| Ps_2823 | 192 | No hits found |  |  | 1 |  |
| Additional file 4 (*continued*) | | | | | | |
| Uniseqs | Length(bp) | Description | Species | E value | No.of clones | Organism |
| Ps_2832 | 336 | No hits found |  |  | 1 |  |
| Ps_2842 | 331 | No hits found |  |  | 1 |  |
| Ps_2846 | 314 | No hits found |  |  | 1 |  |
| Ps_2851 | 399 | No hits found |  |  | 1 |  |
| Ps_2853 | 343 | No hits found |  |  | 1 |  |
| Ps_2855 | 415 | No hits found |  |  | 1 |  |
| Ps_2858 | 364 | No hits found |  |  | 1 |  |
| Ps_2859 | 760 | No hits found |  |  | 1 |  |
| Ps_2866 | 290 | No hits found |  |  | 1 |  |
| Ps_2871 | 695 | No hits found |  |  | 1 |  |
| Ps_2873 | 516 | No hits found |  |  | 1 |  |
| Ps_2876 | 467 | No hits found |  |  | 1 |  |
| Ps_2877 | 598 | No hits found |  |  | 1 |  |
| Ps_2878 | 317 | No hits found |  |  | 1 |  |
| Ps_2880 | 387 | hypothetical protein UM02440.1 | *Ustilago maydis* | 4E-25 | 1 | filamentous fungus |
| Ps_2883 | 325 | No hits found |  |  | 1 |  |
| Ps_2884 | 160 | No hits found |  |  | 1 |  |
| Ps_2897 | 331 | No hits found |  |  | 1 |  |
| Ps_2903 | 663 | No hits found |  |  | 1 |  |
| Ps_2905 | 179 | No hits found |  |  | 1 |  |
| Ps_2911 | 326 | No hits found |  |  | 1 |  |
| Ps_2919 | 342 | No hits found |  |  | 1 |  |
| Ps_2927 | 360 | No hits found |  |  | 1 |  |
| Additional file 4 (*continued*) | | | | | | |
| Uniseqs | Length(bp) | Description | Species | E value | No.of clones | Organism |
| Ps_2945 | 440 | No hits found |  |  | 1 |  |
| Ps_2981 | 272 | No hits found |  |  | 1 |  |
| Ps_2982 | 296 | No hits found |  |  | 1 |  |
| Ps_2989 | 497 | No hits found |  |  | 1 |  |
| Ps_3004 | 317 | No hits found |  |  | 1 |  |
| Ps_3013 | 388 | hypothetical protein UM00142.1 | *Ustilago maydis* | 4E-07 | 1 | filamentous fungus |
| Ps_3023 | 277 | No hits found |  |  | 1 |  |
| Ps_3033 | 386 | No hits found |  |  | 1 |  |
| Ps_3050 | 678 | hypothetical protein UM05510.1 | *Ustilago maydis* | 6E-19 | 1 | filamentous fungus |
| Ps_3083 | 492 | expressed protein | *Cryptococcus neoformans* | 2E-08 | 1 | yeast |
| Ps_3093 | 361 | No hits found |  |  | 1 |  |
| Ps_3100 | 274 | No hits found |  |  | 1 |  |
| Ps_3105 | 375 | No hits found |  |  | 1 |  |
| Ps_3111 | 362 | No hits found |  |  | 1 |  |
| Ps_3119 | 307 | No hits found |  |  | 1 |  |
| Ps_3132 | 608 | No hits found |  |  | 1 |  |
| Ps_3137 | 677 | No hits found |  |  | 1 |  |
| Ps_3167 | 625 | No hits found |  |  | 1 |  |
| Ps_3171 | 311 | No hits found |  |  | 1 |  |
| Ps_3186 | 155 | No hits found |  |  | 1 |  |
| Ps_3189 | 319 | No hits found |  |  | 1 |  |
| Ps_3190 | 582 | conserved expressed protein | *Cryptococcus neoformans* | 4E-12 | 1 | yeast |
| Ps_3212 | 319 | No hits found |  |  | 1 |  |
| Additional file 4 (*continued*) | | | | | | |
| Uniseqs | Length(bp) | Description | Species | E value | No.of clones | Organism |
| Ps_3266 | 355 | No hits found |  |  | 1 |  |
| Ps_3301 | 323 | No hits found |  |  | 1 |  |
| Ps_3321 | 368 | No hits found |  |  | 1 |  |
| Ps_3334 | 549 | No hits found |  |  | 1 |  |
| Ps_3336 | 374 | No hits found |  |  | 1 |  |
| Ps_3345 | 362 | No hits found |  |  | 1 |  |
| Ps_3347 | 329 | No hits found |  |  | 1 |  |
| Ps_3349 | 317 | No hits found |  |  | 1 |  |
| Ps_3359 | 497 | No hits found |  |  | 1 |  |
| Ps_3371 | 156 | No hits found |  |  | 1 |  |
| Ps_3385 | 285 | No hits found |  |  | 1 |  |
| Ps_3392 | 287 | No hits found |  |  | 1 |  |
| Ps_3393 | 275 | No hits found |  |  | 1 |  |
| Ps_3431 | 588 | hypothetical protein AN8834.2 | *Aspergillus nidulans* | 9E-07 | 1 | filamentous fungus |
| Ps_3453 | 673 | No hits found |  |  | 1 |  |
| Ps_3469 | 338 | No hits found |  |  | 1 |  |
| Ps_3470 | 344 | No hits found |  |  | 1 |  |
| Ps_3484 | 320 | No hits found |  |  | 1 |  |
| Ps_3490 | 343 | No hits found |  |  | 1 |  |
| Ps_3498 | 295 | No hits found |  |  | 1 |  |
| Ps_3501 | 318 | No hits found |  |  | 1 |  |
| Ps_3507 | 340 | No hits found |  |  | 1 |  |
| Ps_3510 | 406 | hypothetical protein | *Oryza sativa* | 2E-08 | 1 | plant |
| Additional file 4 (*continued*) | | | | | | |
| Uniseqs | Length(bp) | Description | Species | E value | No.of clones | Organism |
| Ps_3515 | 322 | No hits found |  |  | 1 |  |
| Ps_3525 | 337 | No hits found |  |  | 1 |  |
| Ps_3529 | 171 | hypothetical protein XP_580193 | *Rattus norvegicus* | 1E-07 | 1 | animal |
| Ps_3531 | 224 | No hits found |  |  | 1 |  |
| Ps_3533 | 223 | No hits found |  |  | 1 |  |
| Ps_3555 | 185 | hypothetical protein XP_580193 | *Rattus norvegicus* | 1E-06 | 1 | animal |
| Ps_3566 | 317 | No hits found |  |  | 1 |  |
| Ps_3572 | 658 | No hits found |  |  | 1 |  |
| Ps_3574 | 728 | No hits found |  |  | 1 |  |
| Ps_3575 | 696 | hypothetical protein UM06036.1 | *Ustilago maydis* | 4E-18 | 1 | filamentous fungus |
| Ps_3582 | 385 | No hits found |  |  | 1 |  |
| Ps_3584 | 721 | No hits found |  |  | 1 |  |
| Ps_3610 | 177 | No hits found |  |  | 1 |  |
| Ps_3619 | 242 | No hits found |  |  | 1 |  |
| Ps_3622 | 408 | No hits found |  |  | 1 |  |
| Ps_3630 | 199 | hypothetical protein XP_580193 | *Rattus norvegicus* | 1E-10 | 1 | animal |
| Ps_3634 | 346 | No hits found |  |  | 1 |  |
| Ps_3644 | 212 | No hits found |  |  | 1 |  |
| Ps_3677 | 653 | No hits found |  |  | 1 |  |
| Ps_3682 | 318 | No hits found |  |  | 1 |  |
| Ps_3684 | 301 | No hits found |  |  | 1 |  |
| Ps_3687 | 318 | No hits found |  |  | 1 |  |
| Ps_3690 | 235 | No hits found |  |  | 1 |  |
| Additional file 4 (*continued*) | | | | | | |
| Uniseqs | Length(bp) | Description | Species | E value | No.of clones | Organism |
| Ps_3697 | 638 | No hits found |  |  | 1 |  |
| Ps_3698 | 300 | No hits found |  |  | 1 |  |
| Ps_3703 | 253 | No hits found |  |  | 1 |  |
| Ps_3712 | 311 | No hits found |  |  | 1 |  |
| Ps_3738 | 245 | No hits found |  |  | 1 |  |
| Ps_3758 | 308 | No hits found |  |  | 1 |  |
| Ps_3771 | 503 | hypothetical protein UM00145.1 | *Ustilago maydis* | 3E-26 | 1 | filamentous fungus |
| Ps_3772 | 591 | No hits found |  |  | 1 |  |
| Ps_3773 | 446 | hypothetical protein XP_580193 | *Rattus norvegicus* | 1E-10 | 1 | animal |
| Ps_3775 | 406 | hypothetical protein | *Oryza sativa* | 2E-08 | 1 | plant |
| Ps_3781 | 418 | No hits found |  |  | 1 |  |
| Ps_3789 | 325 | No hits found |  |  | 1 |  |
| Ps_3793 | 647 | No hits found |  |  | 1 |  |
| Ps_3800 | 347 | No hits found |  |  | 1 |  |
| Ps_3801 | 399 | hypothetical protein | *Oryza sativa* | 2E-08 | 1 | plant |
| Ps_3806 | 264 | No hits found |  |  | 1 |  |
| Ps_3807 | 336 | No hits found |  |  | 1 |  |
| Ps_3835 | 407 | No hits found |  |  | 1 |  |
| Ps_3857 | 415 | No hits found |  |  | 1 |  |
| Ps_3867 | 376 | unnamed protein product | *Kluyveromyces lactis* | 1E-14 | 1 | yeast |
| Ps_3875 | 318 | No hits found |  |  | 1 |  |
| Ps_3880 | 621 | unnamed protein product | *Aspergillus oryzae* | 3E-08 | 1 | filamentous fungus |
| Ps_3907 | 430 | No hits found |  |  | 1 |  |
| Additional file 4 (*continued*) | | | | | | |
| Uniseqs | Length(bp) | Description | Species | E value | No.of clones | Organism |
| Ps_3912 | 197 | No hits found |  |  | 1 |  |
| Ps_3913 | 389 | No hits found |  |  | 1 |  |
| Ps_3931 | 372 | No hits found |  |  | 1 |  |
| Ps_3934 | 635 | No hits found |  |  | 1 |  |
| Ps_3940 | 300 | No hits found |  |  | 1 |  |
| Ps_3947 | 448 | No hits found |  |  | 1 |  |
| Ps_3948 | 407 | No hits found |  |  | 1 |  |
| Ps_3950 | 344 | No hits found |  |  | 1 |  |
| Ps_3963 | 565 | No hits found |  |  | 1 |  |
| Ps_3972 | 192 | No hits found |  |  | 1 |  |
| Ps_3981 | 236 | No hits found |  |  | 1 |  |
| Ps_3998 | 372 | unnamed protein product | *Kluyveromyces lactis* | 1E-14 | 1 |  |
| Ps_4033 | 401 | No hits found |  |  | 1 | yeast |
| Ps_4035 | 241 | No hits found |  |  | 1 |  |
| Ps_4044 | 670 | conserved hypothetical protein | *Chaetomium globosum* | 1E-84 | 1 | filamentous fungus |
| Ps_4052 | 626 | hypothetical protein DEHA0E17017g | *Debaryomyces hansenii* | 4E-08 | 1 | yeast |
| Ps_4056 | 569 | unknown | *Arabidopsis thaliana* | 9E-07 | 1 | plant |
| Ps_4063 | 665 | expressed protein | *Cryptococcus neoformans* | 2E-15 | 1 | yeast |
| Ps_4081 | 649 | No hits found |  |  | 1 |  |
| Ps_4099 | 712 | No hits found |  |  | 1 |  |
| Ps_4105 | 726 | No hits found |  |  | 1 |  |
| Ps_4122 | 504 | No hits found |  |  | 1 |  |
| Ps_4145 | 557 | No hits found |  |  | 1 |  |
| Additional file 4 (*continued*) | | | | | | |
| Uniseqs | Length(bp) | Description | Species | E value | No.of clones | Organism |
| zPs_4152 | 409 | No hits found |  |  | 1 |  |
| Ps_4175 | 312 | No hits found |  |  | 1 |  |
| Ps_4178 | 370 | No hits found |  |  | 1 |  |
| Ps_4189 | 324 | No hits found |  |  | 1 |  |
| Ps_4201 | 257 | No hits found |  |  | 1 |  |
| Ps_4204 | 556 | No hits found |  |  | 1 |  |
| Ps_4210 | 631 | hypothetical protein CNBE3260 | *Cryptococcus neoformans* | 6E-26 | 1 | yeast |
| Ps_4219 | 469 | No hits found |  |  | 1 |  |
| Ps_4239 | 689 | No hits found |  |  | 1 |  |
| Ps_4242 | 250 | No hits found |  |  | 1 |  |
| Ps_4254 | 317 | No hits found |  |  | 1 |  |
| Ps_4259 | 363 | No hits found |  |  | 1 |  |
| Ps_4272 | 315 | No hits found |  |  | 1 |  |
| Ps_4279 | 436 | No hits found |  |  | 1 |  |
| Ps_4283 | 641 | No hits found |  |  | 1 |  |
| Ps_4284 | 364 | No hits found |  |  | 1 |  |
| Ps_4286 | 581 | No hits found |  |  | 1 |  |
| Ps_4289 | 429 | No hits found |  |  | 1 |  |
| Ps_4292 | 316 | No hits found |  |  | 1 |  |
| Ps_4301 | 660 | unnamed protein product | *Kluyveromyces lactis* | 6E-14 | 1 | yeast |
| Ps_4308 | 663 | No hits found |  |  | 1 |  |
| Ps_4312 | 397 | No hits found |  |  | 1 |  |
| Ps_4316 | 322 | No hits found |  |  | 1 |  |
| Additional file 4 (*continued*) | | | | | | |
| Uniseqs | Length(bp) | Description | Species | E value | No.of clones | Organism |
| Ps_4318 | 492 | No hits found |  |  | 1 |  |
| Ps_4322 | 422 | No hits found |  |  | 1 |  |
| Ps_4328 | 291 | No hits found |  |  | 1 |  |
| Ps_4329 | 769 | No hits found |  |  | 1 |  |
| Ps_4334 | 324 | No hits found |  |  | 1 |  |
| Ps_4338 | 659 | conserved hypothetical protein | *Cryptococcus neoformans* | 7E-07 | 1 | yeast |
| Ps_4340 | 334 | No hits found |  |  | 1 |  |
| Ps_4345 | 749 | No hits found |  |  | 1 |  |
| Ps_4348 | 321 | No hits found |  |  | 1 |  |
| Ps_4349 | 305 | No hits found |  |  | 1 |  |
| Ps_4355 | 449 | No hits found |  |  | 1 |  |
| Ps_4364-2 | 318 | No hits found |  |  | 1 |  |
| Ps_4369 | 526 | No hits found |  |  | 1 |  |
| Ps_4372 | 530 | hypothetical protein UM04837.1 | *Ustilago maydis* | 8E-06 | 1 | filamentous fungus |
| Ps_4379 | 597 | conserved hypothetical protein | *Cryptococcus neoformans* | 9E-39 | 1 | yeast |
| Ps_4384-1 | 326 | No hits found |  |  | 1 |  |
| Ps_4388 | 244 | No hits found |  |  | 1 |  |
| Ps_4391 | 308 | No hits found |  |  | 1 |  |
| Ps_4417 | 640 | No hits found |  |  | 1 |  |
| Ps_4419 | 626 | No hits found |  |  | 1 |  |
| Ps_4442 | 407 | No hits found |  |  | 1 |  |
| Ps_4445 | 311 | No hits found |  |  | 1 |  |
| Ps_4447 | 322 | No hits found |  |  | 1 |  |
| Additional file 4 (*continued*) | | | | | | |
| Uniseqs | Length(bp) | Description | Species | E value | No.of clones | Organism |
| Ps_4452 | 347 | No hits found |  |  | 1 |  |
| Ps_4460 | 385 | No hits found |  |  | 1 |  |
| zPs_4478 | 266 | hypothetical protein XP_580193 | *Rattus norvegicus* | 1E-10 | 1 | animal |
| Ps_4490 | 506 | hypothetical protein UM04028.1 | *Ustilago maydis* | 1E-12 | 1 | filamentous fungus |
| Ps_4527 | 319 | No hits found |  |  | 1 |  |
| Ps_4533 | 258 | No hits found |  |  | 1 |  |
| Ps_4554 | 552 | No hits found |  |  | 1 |  |
| Ps_4560 | 357 | No hits found |  |  | 1 |  |
| Ps_4564 | 228 | No hits found |  |  | 1 |  |
| Ps_4565 | 167 | No hits found |  |  | 1 |  |
| Ps_4580 | 616 | No hits found |  |  | 1 |  |
| Ps_4581 | 245 | No hits found |  |  | 1 |  |
| Ps_4582 | 176 | hypothetical protein XP_580193 | *Rattus norvegicus* | 2E-07 | 1 | animal |
| Ps_4587 | 687 | No hits found |  |  | 1 |  |
| Ps_4607 | 309 | No hits found |  |  | 1 |  |
| Ps_4610 | 378 | No hits found |  |  | 1 |  |
| Ps_4611 | 266 | No hits found |  |  | 1 |  |
| Ps_4615 | 197 | No hits found |  |  | 1 |  |
| Ps_4621 | 383 | No hits found |  |  | 1 |  |
| Ps_4626 | 218 | No hits found |  |  | 1 |  |
| Ps_4634 | 329 | No hits found |  |  | 1 |  |
| Ps_4643 | 289 | No hits found |  |  | 1 |  |
| Ps_4647 | 243 | No hits found |  |  | 1 |  |
| Additional file 4 (*continued*) | | | | | | |
| Uniseqs | Length(bp) | Description | Species | E value | No.of clones | Organism |
| Ps_4649 | 196 | No hits found |  |  | 1 |  |
| Ps_4655 | 314 | No hits found |  |  | 1 |  |
| Ps_4675 | 256 | No hits found |  |  | 1 |  |
| Ps_4684 | 600 | hypothetical protein UM05130.1 | *Ustilago maydis* | 2E-28 | 1 | filamentous fungus |
| Ps_4701 | 581 | No hits found |  |  | 1 |  |
| Ps_4728 | 351 | No hits found |  |  | 1 |  |
| Ps_4729 | 586 | No hits found |  |  | 1 |  |
| Ps_4740 | 603 | No hits found |  |  | 1 |  |
| Ps_4750 | 434 | hypothetical protein UM02719.1 | *Ustilago maydis* | 9E-20 | 1 | filamentous fungus |
| Ps_4798 | 326 | No hits found |  |  | 1 |  |
| Ps_4834 | 580 | hypothetical protein AN7480.2 | *Aspergillus nidulans* | 6E-51 | 1 | filamentous fungus |
| Ps_4893 | 238 | No hits found |  |  | 1 |  |
| Ps_4896 | 558 | hypothetical protein CNBG2590 | *Cryptococcus neoformans* | 2E-07 | 1 | yeast |
| Ps_4927 | 288 | No hits found |  |  | 1 |  |
| Ps_4934 | 635 | No hits found |  |  | 1 |  |
| Ps_4939 | 643 | hypothetical protein UM02063.1 | *Ustilago maydis* | 9E-33 | 1 | filamentous fungus |
| Ps_4942 | 363 | No hits found |  |  | 1 |  |
| Ps_4950 | 165 | SJCHGC01393 protein | *Schistosoma japonicum* | 9E-06 | 1 | animal |
| Ps_4957 | 293 | No hits found |  |  | 1 |  |
| Ps_4959 | 467 | No hits found |  |  | 1 |  |
| Ps_4966 | 632 | No hits found |  |  | 1 |  |
| Ps_4977 | 518 | Im:7137515 protein | *Danio rerio* | 5E-52 | 1 | animal |
| Ps_4981 | 668 | No hits found |  |  | 1 |  |
| Additional file 4 (*continued*) | | | | | | |
| Uniseqs | Length(bp) | Description | Species | E value | No.of clones | Organism |
| Ps_5001 | 306 | No hits found |  |  | 1 |  |
| Ps_5004 | 523 | No hits found |  |  | 1 |  |
| Ps_5006 | 342 | No hits found |  |  | 1 |  |
| Ps_5017 | 358 | No hits found |  |  | 1 |  |
| Ps_5022 | 705 | No hits found |  |  | 1 |  |
| Ps_5028 | 243 | No hits found |  |  | 1 |  |
| Ps_5054 | 343 | No hits found |  |  | 1 |  |
| Ps_5060 | 554 | No hits found |  |  | 1 |  |
| Ps_5062 | 638 | No hits found |  |  | 1 |  |
| Ps_5063 | 683 | No hits found |  |  | 1 |  |
| Ps_5080 | 318 | unnamed protein product | *Kluyveromyces lactis* | 2E-10 | 1 | yeast |
| Ps_5085 | 353 | No hits found |  |  | 1 |  |
| Ps_5105 | 262 | No hits found |  |  | 1 |  |
| Ps_5116 | 303 | No hits found |  |  | 1 |  |
| Ps_5121 | 554 | No hits found |  |  | 1 |  |
| Ps_5133 | 501 | No hits found |  |  | 1 |  |
| Ps_5138 | 710 | hypothetical protein UM05359.1 | *Ustilago maydis* | 1E-31 | 1 | filamentous fungus |
| Ps_5143 | 134 | No hits found |  |  | 1 |  |
| Ps_5153 | 377 | No hits found |  |  | 1 |  |
| Ps_5154 | 284 | No hits found |  |  | 1 |  |
| Ps_5160 | 269 | No hits found |  |  | 1 |  |
| Ps_5162 | 305 | No hits found |  |  | 1 |  |
| Ps_5165 | 456 | No hits found |  |  | 1 |  |
| Additional file 4 (*continued*) | | | | | | |
| Uniseqs | Length(bp) | Description | Species | E value | No.of clones | Organism |
| Ps_5178 | 333 | similar to hypothetical protein | *Rattus norvegicus* | 1E-19 | 1 | animal |
| Ps_5181 | 338 | unnamed protein product | *Kluyveromyces lactis* | 6E-08 | 1 | yeast |
| Ps_5190 | 371 | No hits found |  |  | 1 |  |
| Ps_5213 | 315 | hypothetical protein XP_580193 | *Rattus norvegicus* | 5E-10 | 1 | animal |
| Ps_5239 | 653 | No hits found |  |  | 1 |  |
| Ps_5246 | 634 | hypothetical protein CNH02200 | *Cryptococcus neoformans* | 5E-13 | 1 | yeast |
| Ps_5251 | 609 | No hits found |  |  | 1 |  |
| Ps_5254 | 582 | No hits found |  |  | 1 |  |
| Ps_5256 | 371 | No hits found |  |  | 1 |  |
| Ps_5269 | 403 | hypothetical protein | *Oryza sativa* | 1E-08 | 1 | plant |
| Ps_5271 | 571 | No hits found |  |  | 1 |  |
| Ps_5272 | 735 | No hits found |  |  | 1 |  |
| Ps_5281 | 450 | No hits found |  |  | 1 |  |
| Ps_5288 | 611 | No hits found |  |  | 1 |  |
| Ps_5314 | 504 | hypothetical protein MGG_ch7g614 | *Magnaporthe grisea* | 2E-10 | 1 | filamentous fungus |
| Ps_5339 | 583 | No hits found |  |  | 1 |  |
| Ps_5347 | 591 | No hits found |  |  | 1 |  |
| Ps_5351 | 179 | hypothetical protein XP_580193 | *Rattus norvegicus* | 8E-07 | 1 | animal |
| Ps_5361 | 206 | No hits found |  |  | 1 |  |
| Ps_5364 | 352 | unknown | *Saccharomyces cerevisiae* | 3E-08 | 1 | yeast |
| Ps_5365 | 521 | hypothetical protein UM04345.1 | *Ustilago maydis* | 3E-21 | 1 | filamentous fungus |
| Ps_5373 | 299 | No hits found |  |  | 1 |  |
| Ps_5375 | 331 | No hits found |  |  | 1 |  |
| Additional file 4 (*continued*) | | | | | | |
| Uniseqs | Length(bp) | Description | Species | E value | No.of clones | Organism |
| Ps_5378 | 267 | No hits found |  |  | 1 |  |
| Ps_5379 | 330 | No hits found |  |  | 1 |  |
| Ps_5381 | 267 | No hits found |  |  | 1 |  |
| Ps_5385 | 683 | No hits found |  |  | 1 |  |
| Ps_5390 | 271 | No hits found |  |  | 1 |  |
| Ps_5393 | 317 | No hits found |  |  | 1 |  |
| Ps_5394 | 344 | No hits found |  |  | 1 |  |
| Ps_5397 | 342 | unnamed protein product | *Kluyveromyces lactis* | 3E-08 | 1 | yeast |
| Ps_5398 | 394 | No hits found |  |  | 1 |  |
| Ps_5428 | 420 | hypothetical protein | *Oryza sativa* | 2E-08 | 1 | plant |
| Ps_5447 | 352 | hypothetical protein XP_580193 | *Rattus norvegicus* | 1E-10 | 1 | animal |
| Ps_5453 | 423 | unnamed protein product | *Kluyveromyces lactis* | 2E-08 | 1 | yeast |
| Ps_5454 | 650 | hypothetical protein DEHA0B10934g | *Debaryomyces hansenii* | 1E-07 | 1 | yeast |
| Ps_5459 | 558 | No hits found |  |  | 1 |  |
| Ps_5488 | 380 | No hits found |  |  | 1 |  |
| Ps_5505 | 562 | No hits found |  |  | 1 |  |
| Ps_5507 | 313 | No hits found |  |  | 1 |  |
| Ps_5511 | 284 | No hits found |  |  | 1 |  |
| Ps_5519 | 338 | No hits found |  |  | 1 |  |
| Ps_5530 | 282 | No hits found |  |  | 1 |  |
| Ps_5535 | 264 | No hits found |  |  | 1 |  |
| Ps_5536 | 263 | No hits found |  |  | 1 |  |
| Ps_5548 | 423 | unnamed protein product | *Kluyveromyces lactis* | 6E-10 | 1 | yeast |
| Additional file 4 (*continued*) | | | | | | |
| Uniseqs | Length(bp) | Description | Species | E value | No.of clones | Organism |
| Ps_5549 | 274 | No hits found |  |  | 1 |  |
| Ps_5551 | 278 | No hits found |  |  | 1 |  |
| Ps_5555 | 415 | hypothetical protein | *Oryza sativa* | 2E-08 | 1 | plant |
| Ps_5561 | 672 | No hits found |  |  | 1 |  |
| Ps_5572 | 317 | No hits found |  |  | 1 |  |
| Ps_5575 | 426 | unnamed protein product | *Kluyveromyces lactis* | 6E-10 | 1 | yeast |
| Ps_5578 | 438 | unnamed protein product | *Kluyveromyces lactis* | 2E-08 | 1 | yeast |
| Ps_5592 | 432 | No hits found |  |  | 1 |  |
| Ps_5596 | 669 | hypothetical protein UM06109.1 | *Ustilago maydis* | 4E-28 | 1 | filamentous fungus |
| Ps_5606 | 319 | No hits found |  |  | 1 |  |
| Ps_5608 | 173 | unknown | *Saccharomyces cerevisiae* | 1E-08 | 1 | yeast |
| Ps_5609 | 389 | No hits found |  |  | 1 |  |
| Ps_5611 | 172 | No hits found |  |  | 1 |  |
| Ps_5613 | 181 | hypothetical protein XP_580193 | *Rattus norvegicus* | 2E-10 | 1 | animal |
| Ps_5615 | 372 | No hits found |  |  | 1 |  |
| Ps_5626 | 191 | hypothetical protein XP_580193 | *Rattus norvegicus* | 8E-08 | 1 | animal |
| Ps_5628 | 107 | No hits found |  |  | 1 |  |
| Ps_5630 | 474 | No hits found |  |  | 1 |  |
| Ps_5632 | 152 | unknown | *Saccharomyces cerevisiae* | 2E-06 | 1 | yeast |
| Ps_5633 | 159 | hypothetical protein XP_580193 | *Rattus norvegicus* | 8E-07 | 1 | animal |
| Ps_5637 | 155 | unknown | *Saccharomyces cerevisiae* | 6E-06 | 1 | yeast |
| Ps_5641 | 214 | No hits found |  |  | 1 |  |
| Ps_5650 | 426 | unnamed protein product | *Kluyveromyces lactis* | 6E-10 | 1 | yeast |
| Additional file 4 (*continued*) | | | | | | |
| Uniseqs | Length(bp) | Description | Species | E value | No.of clones | Organism |
| Ps_5655 | 313 | No hits found |  |  | 1 |  |
| Ps_5662 | 328 | No hits found |  |  | 1 |  |
| Ps_5683 | 488 | No hits found |  |  | 1 |  |
| Ps_5691 | 406 | No hits found |  |  | 1 |  |
| Ps_5693 | 380 | No hits found |  |  | 1 |  |
| Ps_5706 | 339 | No hits found |  |  | 1 |  |
| Ps_5723 | 593 | hypothetical protein CNBH1840 | *Cryptococcus neoformans* | 2E-17 | 1 | yeast |
| Ps_5727 | 323 | No hits found |  |  | 1 |  |
| Ps_5737 | 443 | No hits found |  |  | 1 |  |
| Ps_5762 | 328 | No hits found |  |  | 1 |  |
| Ps_5767 | 454 | hypothetical protein XP_580193 | *Rattus norvegicus* | 1E-10 | 1 | animal |
| Ps_5771 | 262 | No hits found |  |  | 1 |  |
| Ps_5779 | 281 | No hits found |  |  | 1 |  |
| Ps_5806 | 403 | unnamed protein product | *Kluyveromyces lactis* | 2E-09 | 1 | yeast |
| Ps_5851 | 401 | No hits found |  |  | 1 |  |
| Ps_5879 | 310 | No hits found |  |  | 1 |  |
| Ps_5888 | 404 | hypothetical protein | *Oryza sativa* | 2E-08 | 1 | plant |
| Ps_5889 | 324 | No hits found |  |  | 1 |  |
| Ps_5896 | 434 | hypothetical protein XP_580193 | *Rattus norvegicus* | 1E-10 | 1 | animal |
| Ps_5904 | 751 | No hits found |  |  | 1 |  |
| Ps_5905 | 797 | No hits found |  |  | 1 |  |
| Ps_5910 | 443 | No hits found |  |  | 1 |  |
| Ps_5936 | 402 | hypothetical protein | *Oryza sativa* | 2E-08 | 1 | plant |
| Additional file 4 (*continued*) | | | | | | |
| Uniseqs | Length(bp) | Description | Species | E value | No.of clones | Organism |
| Ps_5937 | 432 | unnamed protein product | *Kluyveromyces lactis* | 6E-10 | 1 | yeast |
| Ps_5959 | 327 | No hits found |  |  | 1 |  |
| Ps_5973 | 725 | No hits found |  |  | 1 |  |
| Ps_5974 | 259 | No hits found |  |  | 1 |  |
| Ps_5983 | 322 | No hits found |  |  | 1 |  |
| Ps_5990 | 441 | No hits found |  |  | 1 |  |
| Ps_5991 | 316 | No hits found |  |  | 1 |  |
| Ps_4222 | 229 | No hits found |  |  | 1 |  |
| Ps_5115 | 478 | No hits found |  |  | 1 |  |
| Ps_1890 | 490 | No hits found |  |  | 1 |  |
| Ps_4507 | 219 | No hits found |  |  | 1 |  |
| Ps_2820 | 452 | No hits found |  |  | 1 |  |
| Ps_5933 | 360 | No hits found |  |  | 1 |  |
| Ps_4663 | 240 | No hits found |  |  | 1 |  |
| Ps_5346 | 272 | No hits found |  |  | 1 |  |
