# Supplementary material for: Stage-specific gene expression during urediniospore germination in Puccinia striiformis f. sp tritici
Source: BMC Genomics. 2008 May 1;9:203. doi: 10.1186/1471-2164-9-203 (PMC2386484; doi:10.1186/1471-2164-9-203)
Supplement: Additional file 5 — Sequences of primers and probes used for qRT-PCR. The primer pairs, probes and reference sequences used for qRT-PCR were listed. [file 1471-2164-9-203-S5.doc]

**Addidional file 5:** Sequences of primers and probes used for qRT-PCR

UnigenesSequences（5’ —3’）Ps85Forward TGCCCAAGCCGGTGTTAReverse AGCTTCCTGAGCTCGAATTCTGProbe FAM-CCACCGACAAACGGTGCCGC-TAMRAPs159Forward GATACCGGCGCAGATGCTReverse GTATGGGAACGACACCACAGACTProbe FAM-CAGAGTTTCAGGCGGGTCCCAACC-TAMRAPs28Forward GCTCAATCCAGTGCTACCGCReverse GCTTGAAATTGAGCGGAAACCProbe FAM-CCCGCGTCAACGAGGTCAACAGT-TAMRAPs259Forward TCCACTTCAATGAACGACCAReverse CTGTGCTGCGCTTGTAACATProbe FAM-GTGTTCACAGCCATACGTTGCCTCTG-TAMRAPs261Forward TCTTGATGAGGACTTCTTCGCReverse GTTGATTTGAGGGTGGTGTGAProbe FAM-GAGATTGCTAGCTTCCACTCCACTGC-TAMRA

Ps87Forward GAGGGCAATGTGTCGATGAAReverse TCGATCAAATGACCTTTTCGATTProbe FAM-CCACTTGCATTCCAAACAAGTTCTGCC-TAMRA*P. striiformis* actinForward AGACCTTCAATGCCCCTGCGTTReverse GTGACACCATCTCCAGAATCCAProbe FAM-GCCGTGCTCTCCTTGTATGCCTC-TAMRA
